# Modelling anti-vaccine sentiment as a cultural pathogen

**DOI:** 10.1017/ehs.2020.17

**Published:** 2020-05-07

**Authors:** Rohan S. Mehta, Noah A. Rosenberg

**Affiliations:** Department of Biology, Stanford University, Stanford, CA 94305, USA

**Keywords:** Compartmental models, cultural evolution, evolutionary epidemiology, vaccination

## Abstract

Culturally transmitted traits that have deleterious effects on health-related traits can be regarded as *cultural pathogens*. A cultural pathogen can produce coupled dynamics with its associated health-related traits, so that understanding the dynamics of a health-related trait benefits from consideration of the dynamics of the associated cultural pathogen. Here, we treat anti-vaccine sentiment as a cultural pathogen, modelling its ‘infection’ dynamics with the infection dynamics of the associated vaccine-preventable disease. In a coupled susceptible–infected–resistant (SIR) model, consisting of an SIR model for the anti-vaccine sentiment and an interacting SIR model for the infectious disease, we explore the effect of anti-vaccine sentiment on disease dynamics. We find that disease endemism is contingent on the presence of the sentiment, and that presence of sentiment can enable diseases to become endemic when they would otherwise have disappeared. Furthermore, the sentiment dynamics can create situations in which the disease suddenly returns after a long period of dormancy. We study the effect of assortative sentiment-based interactions on the dynamics of sentiment and disease, identifying a tradeoff whereby assortative meeting aids the spread of a disease but hinders the spread of sentiment. Our results can contribute to finding strategies that reduce the impact of a cultural pathogen on disease, illuminating the value of cultural evolutionary modelling in the analysis of disease dynamics.

## Graphical abstract

**Figure:**
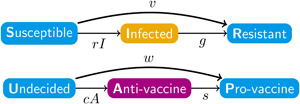

**Media summary:** Coupled contagion model explains how anti-vaccine sentiment, treated as a ‘cultural pathogen’, can promote spread of infectious disease.

## Introduction

Models of cultural evolution examine the dynamics of cultural traits in populations in a manner similar to that of population-genetics studies of the dynamics of genetic variants (Cavalli-Sforza & Feldman, 1981; Boyd & Richerson, 1985; Mesoudi, Whiten, & Laland, 2006). Such models make use of analogous but distinct mechanisms of transmission for cultural and biological characters, as well as analogues of population-genetic processes such as mutation and natural selection.

When a culturally transmitted trait affects the health, reproduction or survival of individuals who carry it, the dynamics of the trait can influence the dynamics of characteristics that are transmitted genetically (Feldman & Laland, 1996; Ross & Richerson, 2014; Creanza et al., 2017) or by other biological mechanisms – such as by the spread of biological pathogens (Tanaka et al., 2002; Epstein et al., 2008; Salathé & Bonhoeffer, 2008; Funk et al., 2009, 2010; Perra et al., 2011; Bauch & Galvani, 2013; Wang et al., 2015). In considering a culturally transmitted trait that has deleterious health effects, we can focus on the concept of a *cultural pathogen*: a culturally transmitted trait whose presence in an individual negatively affects a health-related trait.

In the narrowest sense, we define a cultural pathogen as having a detrimental impact on a specific health-related trait, leaving its impact on other health-related traits unspecified. For example, the concept of a cultural pathogen might be applied to a practice such as ritual eating of the dead in the Fore people of Papua New Guinea, which contributed to transmission of the prion disease kuru (Feldman & Cavalli-Sforza, 1976; Durham, 1982; Lindenbaum, 2008; Whitfield, Pako, Collinge, & Alpers, 2017). The class of cultural pathogens includes premodern medical treatments such as bloodletting, which generally exacerbated rather than alleviated disease conditions and whose continued use was a consequence of cultural transmission in the medical profession (DePalma, Hayes, & Zacharski, 2007; Miton, Claidière, & Mercier, 2015). Persistent folk remedies that have no discernible benefit against diseases they are designed to treat and even cause significant harm – such as the use of harsh chemicals to treat cutaneous leishmaniasis (Ramdas, 2012) and the use of elements such as arsenic, lead and mercury in herbal preparations in traditional Indian medicine (Ernst, 2002; Saper et al., 2008) – are also cultural pathogens (Tanaka, Kendal, & Laland, 2009). Note that our sense of a cultural pathogen is similar to treatments of maladaptive cultural traits in evolutionary models (e.g. Cavalli-Sforza & Feldman, 1981; Boyd & Richerson, 1985); unlike in these treatments, however, we consider the consequences of the cultural trait not on biological fitness but on a specific aspect of health.

The dynamics of social contagions – contagious ideas, information or sentiments (Bauch & Galvani, 2013) – have long been of interest (Goffman & Newill, 1964), possessing many parallels to the dynamics of biological contagions. Among social contagions, the class represented by cultural pathogens generates coupled social and biological dynamics, as dynamics of the cultural pathogen influence those of the associated health-related trait. Thus, to investigate the dynamics of the health-related trait, it is important to consider the dynamics of the cultural pathogen. A coupled perspective can complicate the dynamics of the health-related trait beyond what would be observed in studying its behaviour without the cultural pathogen (Epstein et al., 2008; Funk et al., 2010; Wang et al., 2015).

Anti-vaccine sentiment – an individual predisposition to intentionally avoid vaccination against an infectious disease – is a culturally transmitted trait (Dubé, Vivion, & MacDonald, 2015) that persists endemically in some populations (Gangarosa et al., 1998; Salathé & Khandelwal, 2011; Mellerson et al., 2018; Morrison, Castro, & Meyers, 2020), often despite significant intervention (e.g. Delamater, Leslie, & Yang, 2017). Assuming that a vaccine provides effective protection against an infectious disease, this anti-vaccine sentiment can be regarded as a cultural pathogen in relation to the disease against which the vaccine protects. Not being vaccinated is a risk factor for disease infection; therefore, irrespective of its effects on other behaviours or health-related traits, a culturally transmitted anti-vaccine sentiment satisfies the condition for being a cultural pathogen in relation to the target infectious disease.

Coupled dynamics of anti-vaccine sentiment (a cultural pathogen) and infection by the infectious agent against which the vaccine protects (a biological pathogen) provide a natural example for investigation of coupled contagions. At the same time, analysis of a coupled contagion model can provide insight into the effects of anti-vaccine sentiment dynamics on infectious disease dynamics. Here, we seek to understand the effects of the dynamics of anti-vaccine sentiment on the dynamics of an infectious disease. We build a model of a culturally transmitted anti-vaccine sentiment that acts as a cultural pathogen influencing infection with the disease targeted by the vaccine. We consider a susceptible–infected–resistant (SIR) compartmental model for disease dynamics, coupling it with a separate SIR model for cultural dynamics of the anti-vaccine sentiment.

## Model

Our model concerns two dynamical processes: the spread of an infectious disease and the spread of anti-vaccination sentiment. Disease transmission follows a traditional SIR framework (Kermack & McKendrick, 1932; Hethcote, 2000) with vaccination. We treat anti-vaccination sentiment as a cultural pathogen: by analogy with the SIR model of a transmissible biological pathogen, we model anti-vaccine sentiment as a contagion that can be transmitted to a susceptible individual upon interaction with an ‘infected’ anti-vaccine individual. Transmission of sentiment is analogous to that of an infectious agent and follows an SIR framework in which individuals enter the population susceptible to a contagion and can become sentiment-‘infected’ by interacting with sentiment-infected individuals. Sentiment-infected individuals can ‘recover’ and become pro-vaccine. Pro-vaccine individuals are immune to anti-vaccine sentiment upon further exposure. We treat sentiment-susceptible individuals as undecided about vaccines, and they can become pro-vaccine without having been previously anti-vaccine; this transition represents ‘vaccination’ against the cultural pathogen. Only pro-vaccine individuals can become vaccinated against the disease; this restriction is the mechanism by which the sentiment can facilitate disease spread.

The modelling approach encapsulates phenomena observed in the dynamics of anti-vaccine sentiment. To emphasize the link between the social contagion of anti-vaccination sentiment and the biological contagion of the disease, the model considers sentiment transmission by person-to-person communication, such as by social networks (Brunson, 2013) and social media (Salathé & Khandelwal, 2011; Salathé, Vu, Khandelwal, & Hunter, 2013); it does not consider adoption of anti-vaccine sentiment through other means (Funk et al., 2010; Dubé et al., 2015), such as via individual perception of vaccination and disease risks (Lewis & Speers, 2003; Smailbegovic, Laing, & Bedford, 2003; Wombwell et al., 2015) or interactions between individual-based and social acquisition modes.

The model also accounts for distinctions observed between anti-vaccine and pro-vaccine sentiment. It treats pro-vaccine transitions as individual decisions without social influence, reflecting results that the complex phenomenon of anti-vaccine sentiment (Dubé et al., 2015; Peretti-Watel et al., 2015) behaves more like a contagion than does pro-vaccine sentiment (Salathé et al., 2013). In this framework, pro-vaccine sentiment acts as a cultural inoculation against transmissible anti-vaccine sentiment (McGuire & Papageorgis, 1961; Banas & Rains, 2010) rather than as a transmissible sentiment itself.

To encode the dynamics of the coupled biological and cultural pathogens, we consider a coupled pair of SIR systems, one for disease and one for anti-vaccine sentiment. The population has nine compartments, each corresponding to a pair of classes from the two systems. The disease classes are susceptible (S), infected (I) and resistant (R). The resistant class includes vaccinated individuals. Sentiment classes are undecided (U), anti-vaccine (A) and pro-vaccine (P).

An SIR model with vaccination, vital dynamics (births and deaths), and constant population size *S* + *I* + *R* = 1 has four parameters: birth rate *b*, disease transmission rate *r* for *S* → *I* transitions, disease recovery rate *g* for *I* → *R* transitions, and vaccination rate *v* for *S* → *R* transitions. To maintain a constant population size, we make the standard assumption that birth and death rates are equal, so that the death rate is also *b*. The dynamics follow a system of equations involving time derivatives of the compartments (Hethcote, 2000; Keeling & Rohani, 2007):1
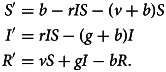


**Figure 1.**
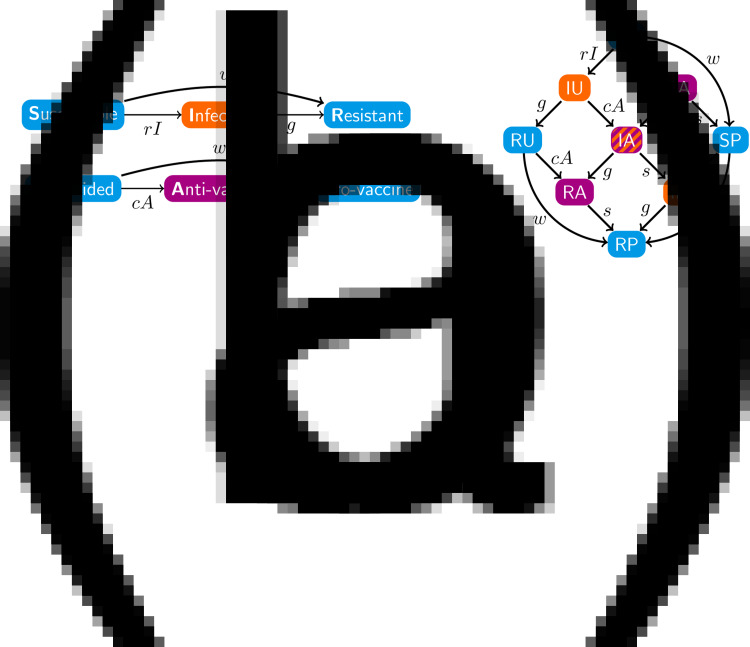
Diagrams of the model. Compartment variable names follow Table 2 and parameter names follow Table 1. (a) The disease and sentiment susceptible–infected–resistant systems shown separately. (b) The full nine-compartment system. An additional parameter *b* describes the rate at which individuals are introduced into the population in compartment *SU* as well as the rate at which individuals are removed from the population in each compartment.

The disease and sentiment components of our model each use the structure of eqn (1). In addition to the disease parameters in eqn (1), our coupled model has three parameters for sentiment dynamics, corresponding to the intercompartmental transition rate parameters *r*, *g* and *v* in the disease model of eqn (1): sentiment transmission rate *c* for *U* → *A* transitions, anti-vaccine-to-pro-vaccine switching rate *s* for *A* → *P* transitions and pro-vaccine decision rate *w* for *U* → *P* transitions. Parameters are summarized in Table 1 and Figure 1a, which displays the two SIR models separately. Unless otherwise stated, we assume that all parameter values are strictly positive.

**Table 1.** Parameters of the model (Figure 1 and eqn 2)

Parameter	Description
*b*	Birth rate
*r*	Disease transmission rate
*g*	Disease recovery rate
*v*	Disease vaccination rate
*c*	Sentiment transmission rate
*s*	Sentiment ‘recovery’ rate
*w*	Sentiment ‘vaccination’ rate

Denoting compartments with two letters, the first for the disease class and the second for the sentiment class (Table 2), dynamics of our model are described by the following system of equations:2
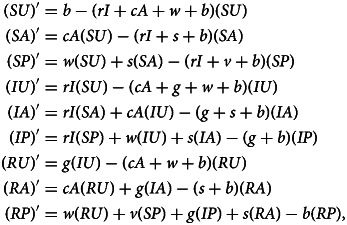
with *SU* + *SA* + *SP* + *IU* + *IA* + *IP* + *RU* + *RA* + *RP* = 1. The two-letter abbreviations represent compartments associated with a specific disease compartment and a specific sentiment compartment. Figure 1b presents a diagram of the full compartmental model described by eqn (2).

**Table 2.** Compartments in the model (Figure 1 and eqn 2). Each entry represents a fraction of the population

		Sentiment state			
		Undecided	Anti-vaccine	Pro-vaccine	Total
Disease state	Susceptible	SU	SA	SP	S
	Infected	IU	IA	IP	I
	Resistant	RU	RA	RP	R
	Total	U	A	P	1

The marginal variables *S*, *I*, *R*, *U*, *A* and *P*, representing the disease-susceptible, disease-infected, disease-recovered, vaccine-undecided, anti-vaccine and pro-vaccine classes, respectively, are defined by summing over the associated compartments:3
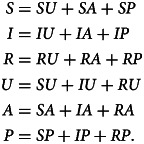


Summing over disease classes and writing eqn (2) in terms of *U*, *A* and *P* yields the SIR model in those variables (eqn 18 in Appendix A – Supplementary Information). We call this system the ‘sentiment subsystem’. Summing over sentiment classes in an analogous way yields the ‘disease subsystem’ (eqn 19 in Appendix A). This system in terms of *S*, *I*, and *R* is close to the standard SIR model (eqn 1), with the only difference being that the vaccination rate *v* is multiplied by a quantity *p* = (*SP*)/*S*, the fraction of *S* individuals that are pro-vaccine and therefore the only individuals that can be vaccinated against the disease. This modification is the source of the coupling between the disease and sentiment subsystems, as the sentiment state affects a transition in the disease state through this modification.

The most important quantity in a standard SIR model is the basic reproduction number *R*_0_ (Hethcote, 2000; Keeling & Rohani, 2007), which determines the equilibrium behaviour of the model. For eqn (1), this quantity is *R*_0_ = *r*/(*g* + *b*). For our model (eqn 2), we have two analogous quantities, one for the disease subsystem (*R*_0_) and one for the sentiment subsystem (*C*_0_):4
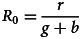
5
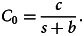


The quantity *R*_0_ (eqn 4) represents the expected number of infected individuals produced by a single disease case, assuming the rest of the population is susceptible to the disease (*S* ≈ 1). The quantity *C*_0_ (eqn 5) represents the expected number of anti-vaccine individuals produced by a single individual with this sentiment, assuming that the rest of the population is undecided (*U* ≈ 1).

These quantities reflect the ability of each contagion to be transmitted, and they play important roles in determining equilibrium behaviour. If *R*_0_ or *C*_0_ exceeds 1, then transmission outpaces recovery for the associated contagion. If *R*_0_ or *C*_0_ is even larger, then transmission might also outpace the effects of vaccination and pro-vaccine decisions. It is only if this additional condition is met that the contagion can maintain itself in the population.

## Results

### Equilibria

We first analyse the equilibrium states. Approach to equilibrium is one possible outcome of the long-term behaviour of a dynamical model with fixed parameter values, and we do not observe any periodic or chaotic behaviour in our model. We find the possible equilibrium states for our model by setting the nine equations in eqn (2) to 0. This procedure yields four solutions that are analogous to considering pairs of equilibrium solutions of the standard SIR model (eqn 1). A standard SIR model has a solution at which the contagion is extinct (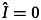) and a solution at which it is endemic (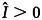). The four equilibria in our model thus correspond to: disease and sentiment both extinct (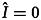, 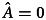), disease extinct and sentiment endemic (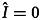, 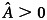), disease endemic and sentiment extinct (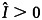, 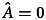) and disease and sentiment both endemic (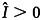, 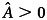).

For almost all possible parameter values, only one equilibrium is stable, meaning that a small deviation from the equilibrium returns to the equilibrium state. The only situations in which multiple equilibria are stable occur if all stable equilibria yield the same vector of nine compartment frequencies. We determined the local linear stability of the four equilibria by studying the eigenvalues of the Jacobian matrix of eqn (2), a standard technique for studying stability in dynamical systems (Beltrami, 1998). See Appendices B–E for details and Table 3 for stability conditions.

**Table 3.** Conditions for local stability of the four equilibria. An equilibrium is stable for a given set of parameter values (*b*, *r*, *g*, *v*, *s*, *c*, *w*) if and only if the conditions for *R*_0_ and *C*_0_ are both met. The columns denoted 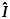 and 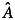 indicate presence (+) or absence (−) of disease and sentiment, respectively, at equilibrium

Equilibrium	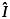	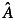	Disease condition	Sentiment condition
Disease- and sentiment-free equilibrium (DSFE)	−	−	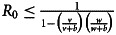	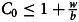
Disease-free equilibrium (DFE)	−	+	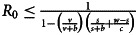	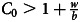
Sentiment-free equilibrium (SFE)	+	−	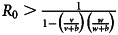	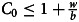
Endemic equilibrium (EE)	+	+	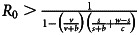	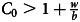

#### DSFE

The first equilibrium, the disease- and sentiment-free equilibrium (DSFE), has both disease and anti-vaccine sentiment extinct (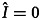, 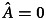). Variables with nonzero equilibrium values are6
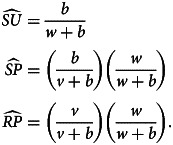


Only susceptible and resistant individuals for both traits are present at the DSFE. The vaccination rate *v* and pro-vaccine decision rate *w*, which together characterize the rate at which disease-susceptible, undecided *SU* individuals move to the pro-vaccine, vaccinated *RP* state, occur together frequently in our equilibrium results and are a reflection of the overall vaccination behaviour in the population. For the DSFE, the equilibrium susceptible population is7
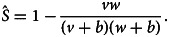
The value of 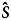 (eqn 7) is driven primarily by the strength of vaccination behaviour in the population. If *v* and *w* are large, then most individuals will be vaccinated and 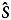 is close to 0.

The DSFE is stable if sentiment transmission does not outpace pro-vaccine decision-making and disease transmission does not outpace vaccination (Table 3).

#### DFE

The second equilibrium, the disease-free equilibrium (DFE), has endemic sentiment but extinct disease (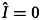, 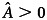). Variables with nonzero equilibrium values are8
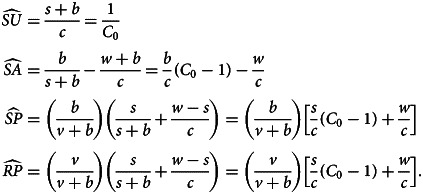
The equilibrium susceptible population for the DFE is9
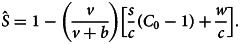


The two terms subtracted from 1 in eqn (9) reflect the two possible routes towards vaccination when sentiment is present (*SU* → *SA* → *SP* → *RP* and *SU* → *SP* → *RP*). The conflicting presence of the sentiment transmission rate *c* in the numerator and denominator of the term (*s*/*c*)(*C*_0_ − 1) (recalling eqn 5 for *C*_0_) reflects the fact that sentiment transmission and sentiment switching undermine each other but that sentiment switching only matters if sentiment is transmitted. In general, if pro-vaccine decisions are fast, sentiment transmission is slow or sentiment switching is fast, then most individuals will be vaccinated. If pro-vaccine decisions are slow, sentiment transmission is fast or sentiment switching is slow, then most individuals will be susceptible.

The DFE is stable if sentiment transmission outpaces pro-vaccine decision-making but disease transmission does not outpace vaccination (Table 3).

#### SFE

The third equilibrium, the sentiment-free equilibrium (SFE), has endemic disease but extinct sentiment (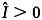, 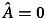). For this equilibrium, it is easiest to state the values of 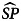 and 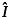 and then write other nonzero values in terms of these two quantities. We have10
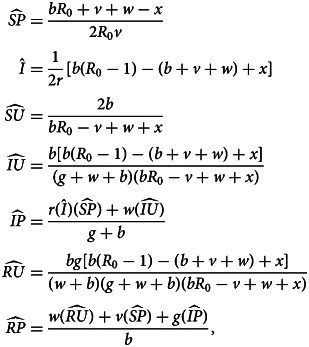
where
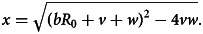


The SFE is stable if sentiment transmission does not outpace pro-vaccine decision-making but disease transmission outpaces vaccination (Table 3).

#### EE

The fourth equilibrium, the endemic equilibrium (EE), has both endemic disease and endemic anti-vaccine sentiment (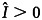, 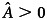). The analytical form of this equilibrium is obtained by considering the real positive root of a quartic equation in 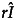 (Appendix A). From 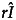, we divide by *r* to obtain 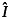, and from 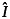, we obtain 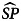:11
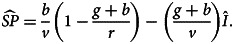
From 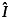 and 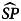, we obtain all other quantities:12
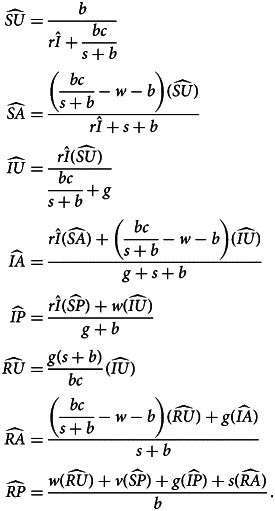


The EE is stable if sentiment transmission outpaces pro-vaccine decision-making and disease transmission outpaces vaccination (Table 3). This equilibrium potentially allows diseases with low transmission rates to be maintained in the population owing to the existence of anti-vaccine sentiment.

### *R*_0_ **and disease endemism**

In the standard SIR model without vaccination (eqn 1, *v* = 0), the basic reproduction number *R*_0_ (eqn 4) is the expected number of secondary cases of a disease produced by a typical infected individual in a population of susceptible individuals. This quantity determines the ultimate fate of the disease: if *R*_0_ < 1, then the disease goes extinct, and if *R*_0_ > 1, then the disease is endemic at equilibrium. With vaccination included in an SIR model with no anti-vaccine sentiment (eqn 1, *v* > 0), the criterion for disease endemism is13
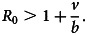
With vaccination present, *R*_0_ can exceed 1, the threshold for disease maintenance in an unvaccinated population, but can fail to exceed 1 + *v*/*b*, so that the disease still goes extinct. By enlarging this threshold for *R*_0_, vaccination makes it more difficult for the disease to persist.

Our model has two conditions for disease endemism, depending on whether sentiment is extinct or endemic. If sentiment is extinct at equilibrium, then the condition for disease endemism is14
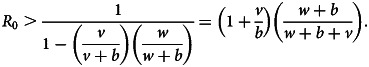
Here, we have rewritten the result from Table 3 to demonstrate that the critical value of *R*_0_, the right-hand side of eqn (14), is always less than or equal to the critical value of *R*_0_ for a system with vaccination but no sentiment dynamics: the right-hand side of eqn (13). No sentiment is present at equilibrium in this case, so the difference between eqns (14) and (13) is due to the presence of undecided, unvaccinated individuals. Their presence hinders the effects of vaccination, as it decreases the value of *R*_0_ that the disease must achieve to be endemic. However, from eqn (14), the critical value of *R*_0_ in this case is still always greater than or equal to 1. Thus, the presence of undecided, unvaccinated individuals resulting from sentiment dynamics maintains an *R*_0_ critical value that lies between that of the model without vaccination and that of the model with vaccination but no sentiment.

If sentiment is endemic at equilibrium, then the condition for disease endemism is15
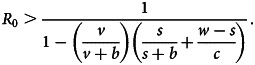
The critical value of *R*_0_, the right-hand side of eqn (15), is less than or equal to the critical value of *R*_0_ in a system with vaccination but no sentiment – the right-hand side of eqn (13) – if *s*/(*s* + *b*) + (*w* − *s*)/*c* ≤ 1. This condition is equivalent to *bc* ≥ (*s* + *b*)(*w* − *s*), an inequality that is satisfied for the case with sentiment endemic at equilibrium: rearranging *C*_0_ > 1 + *w*/*b* yields *bc* > (*s* + *b*)(*w* + *b*) > (*s* + *b*)*w* > (*s* + *b*)(*w* − *s*). Thus, as in the case with extinct sentiment (eqn 14), endemic anti-vaccine sentiment hinders the effects of vaccination by decreasing the critical value of *R*_0_ that the disease must achieve to be endemic. Because all parameters in our model are nonnegative, and because *s*/(*s* + *b*) + (*w* − *s*)/*c* = (*s*/*c*)(*C*_0_ − 1) + *w*/*c* and *C*_0_ > 1, the denominator of the right-hand side of eqn (15) is always less than or equal to 1, so the critical value of *R*_0_ in this case is always greater than or equal to 1. Thus, the effects of endemic sentiment also generate an *R*_0_ critical value between that of the model without vaccination and that of the model with vaccination but no sentiment: the condition for disease spread in a scenario with vaccination and sentiment is stricter than in a model with no vaccination, but not as strict as with vaccination and no sentiment.

The right-hand side of eqn (15) is greater than or equal to that of eqn (14) if and only if *bC*_0_ ≥ *w* + *b*. This condition is already satisfied with endemic sentiment (Table 3), so it is possible for a disease to be endemic if sentiment is endemic when it would not be endemic if sentiment were extinct.

### Effects of parameters on stability of equilibria

**Figure 2.**
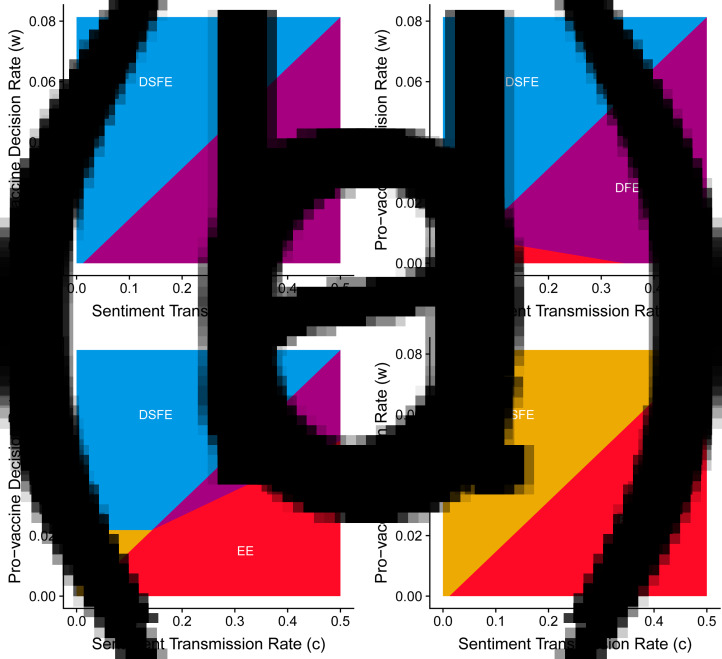
Stability regimes for varying sentiment transmission rate *c* and pro-vaccine decision rate *w*. (a) *R*_0_ ∈ [0, 1]. (b) 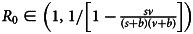. (c) 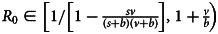. (d) 
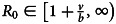
. Parameter values for (a–d) are (*b*, *g*, *v*, *s*) = (0.002, 0.14, 0.14, 0.01). These parameters lead to transitional *R*_0_ values of 
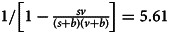
 and 1 + *v*/*b* = 72.42. For (a), *r* = 0.1 so that *R*_0_ = 0.69. For (b), *r* = 0.7 so that *R*_0_ = 4.83. For (c), *r* = 1.5 so that *R*_0_ = 10.36. For (d), *r* = 11 so that *R*_0_ = 75.94. These parameter values were chosen so that the stability regions of all possible equilibria would be visible in each panel.

We now study the effects of individual parameters on the stability of the equilibria. The compound parameters *R*_0_ and *C*_0_ in Table 3 suffice to determine whether or not an equilibrium is stable. To understand the effects of specific parameters, we visualize a subset of the parameter space. In particular, we focus on varying the sentiment parameters *c*, *s* and *w*, keeping the birth rate *b* and the disease parameters *r*, *g* and *v* fixed at values that represent well-studied diseases. As in standard SIR models in which *r*, *g*, and *v* are rate parameters that range from 0 to infinity (Hethcote, 2000), the sentiment transmission rate *c*, the sentiment switching rate *s*, and the pro-vaccine decision rate *w* can range from 0 to infinity. We can therefore plot regions of the first quadrant of a plane considering two of these three variables to depict stability regions for the four equilibria. Figure 2 focuses on the *c*–*w* plane. The boundary curves in Figure 2 are derived in Appendix F.

Note that in our model formulation, one might expect parameters *w*, the pro-vaccine decision rate, and *c*, the sentiment transmission rate, to be inherently inversely related, as a cultural environment with a high pro-vaccine decision rate could be incompatible with high transmissibility of anti-vaccine sentiment. Concurrently high values of *w* and *c* could arise with an underlying polarization in the population on the topic of vaccination, perhaps owing to political fracturing (e.g. Jegede, 2007; Dubé et al., 2015) or the presence of a non-vaccinating religious community (e.g. Woudenberg et al., 2017). Concurrently low values could arise in a situation of general indifference towards vaccines (e.g. Velan, Kaplan, Ziv, Boyko, & Lerner-Geva, 2011; MacDougall & Monnais, 2018). Thus, the two parameters are not necessarily inversely related and it is useful to consider the full *c*–*w* plane.

In Appendix F, it is shown that all parameter sets give rise to one of four regimes for the stability region in the *c*–*w* plane, with the regime determined by the value of *R*_0_. The four cases correspond to four ranges for the value of *R*_0_. The three *R*_0_ values at which the regime transitions from one type to another are discussed in Appendix F.

First, if *R*_0_ < 1, then the disease always goes extinct, and only the DSFE and DFE are possible (Figure 2a). This result is the same as in a standard SIR model, except that increasing the sentiment transmission rate *c* or decreasing the pro-vaccine decision rate *w* results in endemic sentiment.

Next, if
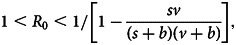
then the disease can spread in a completely susceptible population, and it is possible for the SFE and the EE to be stable in addition to the other two equilibria (Figure 2b). This case is most relevant for large values of the sentiment switching rate *s*, as the range of *R*_0_ values that produce it is narrow for small *s*. As the sentiment transmission rate *c* increases, for large *s*, increased sentiment transmission increases vaccination in the population: many people transition from undecided to anti-vaccine and then, because of the high sentiment switching rate *s*, quickly become pro-vaccine. This situation, in which increased sentiment transmission can move the disease to extinction, represents a disease whose transmissibility cannot overcome the vaccination induced by sentiment switching after transmission of anti-vaccine sentiment.

Different behaviour occurs (Figure 2c) if
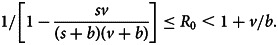


In this range, the disease is transmissible enough to overcome the increased vaccination induced by sentiment switching. Increasing the sentiment transmission rate *c* now makes the disease endemic.

Finally, if 1 + *v*/*b* ≤ *R*_0_, then the disease is transmissible enough to overcome vaccination without assistance from anti-vaccine sentiment. In this situation, the DSFE and DFE are no longer stable anywhere, and decreasing vaccination by decreasing the pro-vaccine decision rate *w* or increasing the sentiment transmission rate *c* first leads to stability for the SFE and then for the EE.

### Disease examples

**Figure 3.**
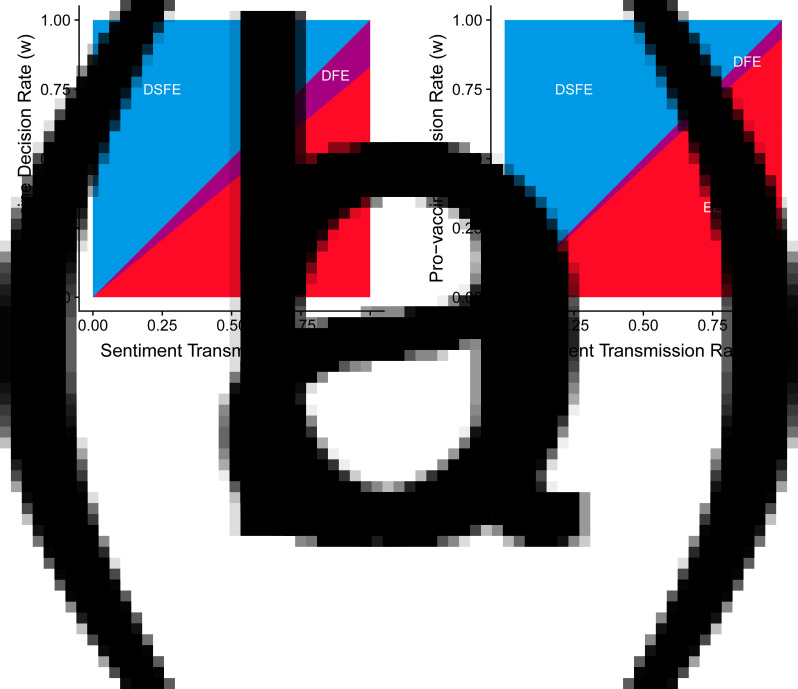
Stability regimes with parameter values chosen to represent two diseases. (a) Polio. (b) Measles. Parameter values are taken from Table 4. These values correspond to Figure 2c, where 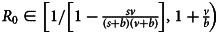. The sentiment-free equilibrium (SFE) is stable and lies in a region of the bottom-left corner of each plot, a region which is too small to see here.

To get a sense for which equilibria are stable in practical settings, we reproduce Figure 2 using disease parameter values that are commonly used to model polio (Figure 3a) and measles (Figure 3b). The parameter values appear in Table 4.

**Table 4.** Parameters for polio and measles. The disease transmission rate *r* is calculated from the basic reproduction number *R*_0_, disease recovery rate *g* and birth rate *b* using eqn (4). Polio *R*_0_, measles *R*_0_ and measles *g* are taken from Anderson and May (1991). Polio *g* is from Tebbens et al. (2005). The value of *b* is from Hamilton et al. (2015). The value *s* = 0 is chosen for simplicity, and we expect *s* to be low in real populations. The value of *v* was chosen so that it takes about 7 days on average for a newly pro-vaccine individual to become vaccinated. The parameters *r*, *g*, *v*, and *s* are reported in units of days^−1^; *b* uses units of per capita births per day. *R*_0_ is dimensionless. The values in the table were used to produce Figure 3

Parameter	Polio	Measles
*R*_0_	6	15
*b*	3.6 × 10^−5^	3.6 × 10^−5^
*r*	0.17	2.14
*g*	0.029	0.14
*v*	0.14	0.14
*s*	0	0

Parameter values for polio reside in case 3 (Figure 2c). Most of the parameter space in Figure 3a consists of regions where either the DSFE or the EE is stable, indicating a tight coupling between disease and sentiment endemism. In a non-trivial region where the DFE is stable, sentiment is endemic, but does not reduce vaccination sufficiently to permit disease to persist. A small region of Figure 3a where the SFE is stable is not visible but exists near the origin.

Parameter values for measles also reside in case 3 (Figure 2c). For measles, with a higher *R*_0_ than polio, the regions in which either the DSFE or the EE is stable now occupy almost the entire parameter space in Figure 3b, indicating extremely tight coupling between disease and sentiment endemism. The high *R*_0_ for measles couples the persistence of the disease to the persistence of sentiment, as disease can only persist in the population owing to sentiment endemism that reduces vaccination sufficiently for the disease to spread. This effect reflects a general result we derive in Appendix F: the greater the value of *R*_0_, the larger the range of parameter values over which the DSFE or the EE is stable and the smaller the range over which the DFE or the SFE is stable.

### Transient oscillations

Thus far, we have been examining equilibria of the model. We now turn our attention to the transient dynamics, solving eqn (2) numerically by specifying initial conditions and using a numerical differential-equation solver (see Appendix G). We begin with a population consisting primarily of susceptible individuals, so that an epidemic occurs early in the dynamics.

**Figure 4.**
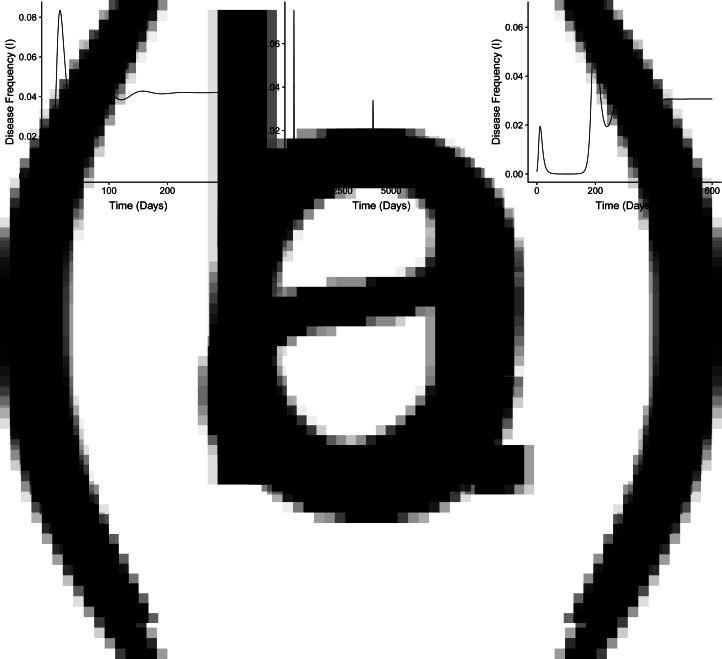
Transient dynamics of the disease frequency for three different parameter sets. (a) Damped oscillation towards the endemic equilibrium (EE). The parameter values are (*b*, *r*, *g*, *v*, *c*, *s*, *w*) = (0.02, 0.8, 0.25, 0.2, 0.8, 0, 0.1). (b) Repeated epidemic spikes separated by long pauses. Parameter values are the same as in (a) except *b* = 0.0002. (c) Two epidemic regimes separated by a long pause during which the disease appears to go extinct. Parameter values are the same as in (a) except *w* = 0.25.

For parameter values that ultimately reach the endemic equilibrium, the transient dynamics are often oscillatory. We have observed two qualitatively different oscillation patterns in the transient dynamics of eqn (2). Figure 4 displays the patterns in three examples with initial compartment frequencies *SU* = 0.998 and *IU* = *SA* = 0.001.

The first pattern (Figure 4a) is a damped oscillation around a stable EE. Comparably to a pattern sometimes seen in a standard SIR model (Wang et al., 2016), the amplitude of oscillation in the frequency of infected individuals becomes negligibly small after only a few cycles. In this example, the oscillatory pattern leads to two noticeable epidemics in a year, with the second less severe, before the disease reaches a relatively high endemic frequency (about 4%). The second pattern (Figure 4b) consists of large epidemics followed by periods in which the disease is nearly extinct. This example differs in that the birth rate *b* is smaller by a factor of 100, slowing the influx of undecided, susceptible individuals to such an extent that the disease frequency can only recover after long periods, when enough susceptible individuals have entered the population. This pattern is also observed in the standard SIR model when the endemic disease frequency is small and the system therefore has parameter values that give rise to the EE but are close to the region that produces the DFE (Wang et al., 2016). In this example, a series of epidemics occurs over a period of years, interrupting periods during which the disease is nearly extinct.

Figure 4c displays a situation that possesses both the damped oscillation around the EE from Figure 4a as well as the long pause between epidemics from Figure 4b. When extended over a longer period, the dynamics in Figure 4b in fact behave similarly to those in Figure 4c, with a damped oscillation to the EE, except that Figure 4b has multiple initial epidemic spikes rather than a single one, and the equilibrium disease frequency is small. Three epidemics occur over the course of a year, with the second more severe than the first and the third less dramatic than the second. During the period between the first two epidemics, the disease appears to be extinct. After the third epidemic, the disease settles into a relatively high endemic frequency (about 3%). In all cases in Figure 4, the disease goes extinct if the initial condition has no anti-vaccine sentiment.

**Figure 5.**
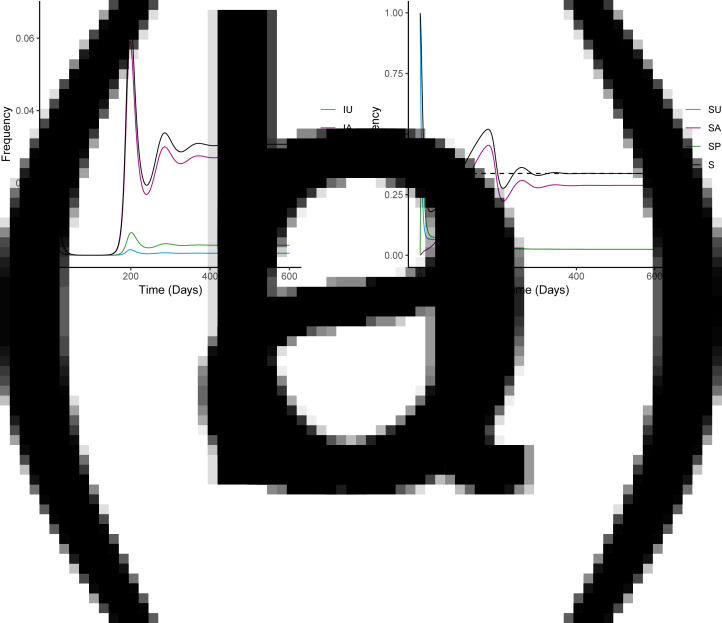
Dynamics of compartments during long-pause oscillation behaviour. (a) Frequencies of disease-infected individuals of all sentiment classes and the total infected frequency. (b) Frequencies of disease-susceptible individuals of all sentiment classes and the total susceptible frequency. The dashed horizontal line is the 1/*R*_0_ threshold frequency of disease-susceptible individuals necessary for the disease to spread. The parameter values follow Figure 4c.

Figure 5 further explores the dynamics of the oscillation seen in Figure 4c, showing the same dynamics but with more compartments. Figure 5a displays the disease-infected compartments *IU*, *IA*, *IP* and their total, *I*. The first epidemic at ~10 days consists of an increase in pro-vaccine *IP* individuals, suggesting that this epidemic is driven by pro-vaccine individuals getting infected with the disease before they have a chance to become vaccinated. This situation occurs because the initial population contains almost all disease-susceptible individuals.

In contrast, the second epidemic consists mostly of *IA* individuals, suggesting that this epidemic is driven by anti-vaccine individuals. The sentiment trajectories in Figure 5b support this claim, as an increase in the disease-susceptible, anti-vaccine compartment *SA* pushes the disease-susceptible frequency *S* above 1/*R*_0_, the threshold necessary for the disease to spread.

### Migration-triggered epidemics

In long pauses between oscillations such as those seen in Figure 4b and c, the disease drops to low frequencies. For instance, in the specific scenario shown in Figure 4c, the lowest disease frequency is 1.2 × 10^−5^. In practical settings, such low values might correspond to extinction of the disease. Thus, because disease prevalence can be effectively 0, we explored scenarios in which a single infected individual is reintroduced to the population.

**Figure 6.**
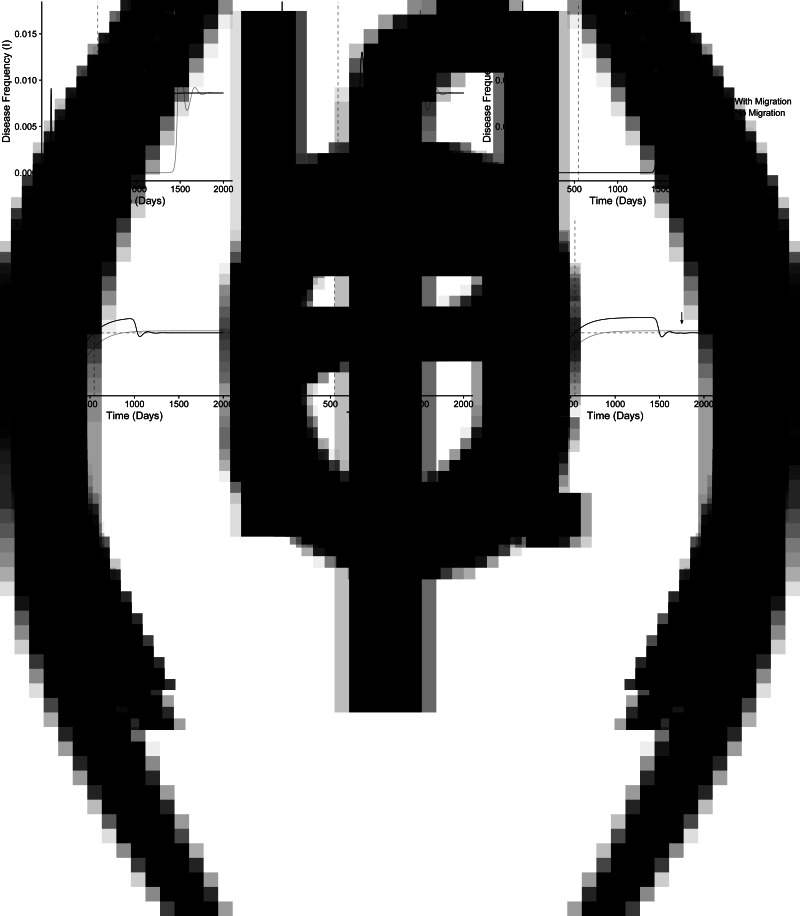
Introducing a single new case of a disease into the population can affect the long-pause oscillation of the disease frequency differently, depending on when it is introduced. (a) Introduction early in the apparent extinction of the disease. (b) Introduction late in the apparent extinction of the disease. (c) Introduction after the disease has re-established and is oscillating towards an endemic equilibrium. (d–f) Disease-susceptible (*S*) and anti-vaccine (*A*) frequencies for the trajectories in (a–c). An arrow indicates the new case. In all panels, for the grey trajectory, provided as a reference, no new case is introduced. The vertical dashed line indicates the point at which disease frequency starts increasing after the initial epidemic, during its apparent extinction. The horizontal dashed line in (d–f) is the 1/*R*_0_ threshold frequency of disease-susceptible individuals necessary for disease spread. Parameters used in this example are the same as in Figure 4c, except *w* is increased to 0.5 to provide a longer pause for illustration.

Figure 6 considers introduction of a new disease case in a population of size 1000. We simulate this event by pausing the numerical solution of our model, instantaneously increasing disease frequency by 0.001, renormalizing the frequencies to sum to 1 and then completing the solution. From a baseline pattern similar to Figure 4c, the effect of this new case depends on when it is introduced. During the interval between the first two epidemics, when the disease is effectively extinct, a critical time exists at which the introduction quickly leads to an epidemic. The critical time is the time at which anti-vaccine sentiment has generated enough unvaccinated individuals that disease frequency would begin increasing in the absence of the new case (indicated by the time at which the frequency of susceptibles crosses the horizontal dashed line in Figures 6d–f).

The grey trajectories in Figure 6a–c show the unperturbed disease frequency over time. In Figure 6a, a new case is introduced at day 250, prior to the critical time point, as the susceptible frequency lies below the threshold value in Figure 6d. The disease does not start epidemics, although the next epidemic does start earlier than without the new case. When the new case appears at day 1750 (Figure 6c), after the new epidemics have commenced and the susceptible frequency lies below the threshold value (Figure 6f), the oscillatory approach to endemic equilibrium is mildly perturbed but recovers quickly, with no lasting effect of the new case. However, when the new case appears at day 750 (Figure 6b), after the critical time point at which anti-vaccine sentiment has generated a sizeable population of unvaccinated individuals – indicated by the concurrent rise in the frequency of anti-vaccine sentiment and disease-susceptible individuals in Figure 6e – the new case immediately triggers epidemics. Thus, even if the disease effectively goes extinct, sentiment dynamics create a situation in which reintroducing a small number of infected individuals initiates new epidemics. In this specific example, such a migration immediately induces an epidemic only after a 15-month delay after the initial epidemic.

### Assortative meeting

Homophily – or the tendency of like to interact with like – is a general property of human social behaviour (McPherson, Smith-Lovin, & Cook, 2001). Vaccination behaviour exhibits homophily, so that anti-vaccine sentiment is not homogeneously distributed in populations (e.g. Salathé & Khandelwal, 2011; Barclay et al., 2014; Onnela et al., 2016; Fu, Zimet, Latkin, & Joseph, 2019). We can extend our main model in eqn (2) to permit homophily through the phenomenon of assortative meeting (Eshel & Cavalli-Sforza, 1982), modifying interaction probabilities by a parameter that increases the probability of interactions between individuals of the same sentiment class (*A* × *A* or *P* × *P*). The goal of this modification is to understand the influence of clustering in the population of anti-vaccine sentiment on the dynamics, as assortative meeting increases contacts among *A* individuals and among *P* individuals, and it decreases contacts between *A* and *P* individuals.

We consider an assortative meeting parameter *α* that affects the result of two draws with replacement from the population. We assume that undecided (*U*) individuals are unaffected by the rate of assortative meeting: if the first individual drawn is undecided (*U*), then the second draw is from the whole population. If the first individual drawn is anti-vaccine (*A*) or pro-vaccine (*P*), however, then the second draw is of type *A* or *P*, respectively, with probability *α* and it is from the whole population with probability 1 − *α*. Thus, *α* = 0 corresponds to the well-mixed case with no assortative meeting (eqn 2), and *α* = 1 corresponds to complete assortative meeting.

The new system of equations with this assortative meeting assumption is:16
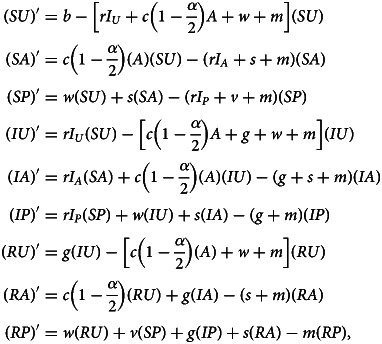
where17
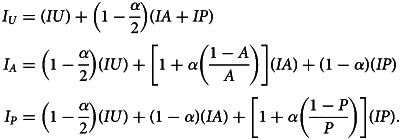


In eqn (17), *I*_*i*_ for *i* = *U*, *A*, *P* represents a modified frequency of infected individuals available for interaction with an individual of sentiment type *i* (Appendix H). All three values reduce to *I* = *IU* + *IA* + *IP* if *α* = 0.

To examine the effect of assortative meeting on our model, we numerically solved eqn (16) using the measles parameters from Table 4 with *c* = 0.8, *w* = 0.14 and *s* = 0 (Appendix G). These values have the same order of magnitude as disease parameters *r* and *v* (Table 4) and lead to an endemic equilibrium (Figure 3). As an initial condition, we used *SU* = 0.06375, *SA* = 0.00525, *IU* = 0.001, *RA* = 0.06975 and *RP* = 0.86025, with other classes set to zero. We chose these initial frequencies to be near the 1 − 1/*R*_0_ threshold for measles, *RA* + *RP* = 93% vaccine coverage, with overall anti-vaccine sentiment frequency *SA* + *RA* = 7.5%, the highest statewide rate of kindergarten non-medical vaccine exemptions in the US during 2017–2018 (Mellerson et al., 2018).

**Figure 7.**
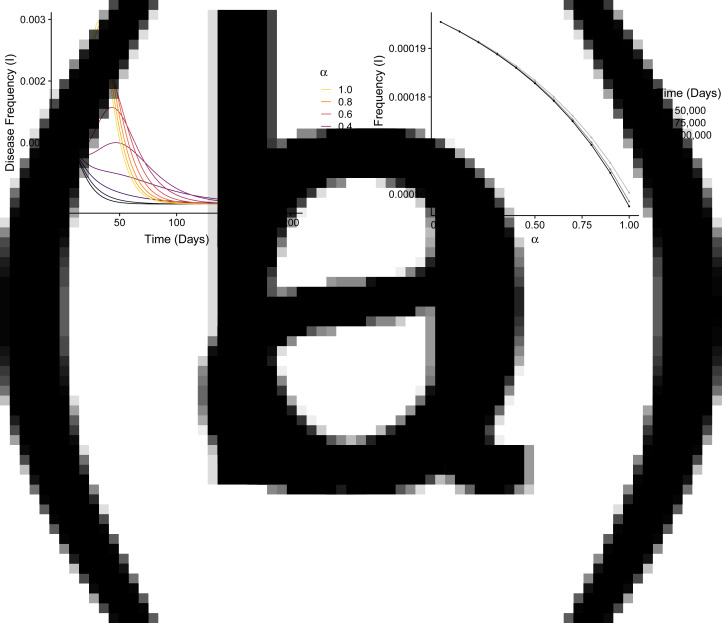
Disease frequency over time with various levels of assortative meeting. (a) Initial behaviour for a system that starts near the herd-immunity level for measles, with 7.5% of individuals having anti-vaccine sentiment. (b) Disease frequency after 50,000, 75,000 and 100,000 days for a system that starts at the endemic equilibrium. Both panels use measles parameters from Table 4 with *c* = 0.8 and *w* = 0.14. These values are chosen so that *c* is the same as that used in other analyses (Figures 4–6), *w* is on the same scale as *v* (Table 4) and the EE is stable (Table 3).

For a variety of values of *α*, Figure 7a shows this solution for the first 200 days, after which the initial epidemic has subsided. We see that a high value of *α* can trigger an epidemic that would otherwise not have occurred at *α* = 0. For high values of *α*, the epidemic is also shorter. Increased assortative meeting spreads disease within the anti-vaccine class, increasing the disease frequency; however, it also hinders both disease transmission to the undecided class and conversion of the undecided class to the anti-vaccine class, so that disease frequency drops quickly.

To study the effects of *α* on the equilibrium frequency of the disease, we numerically solved eqn (16) for 100,000 time steps interpreted as days, using the same parameters as in Figure 7a but starting at the endemic equilibrium for the original *α* = 0 model (eqn 2). The time period of 100,000 days was chosen to be long enough to rule out long-delayed epidemics.

Figure 7b plots the resulting values at 50,000, 75,000 and 100,000 time steps of the disease frequency *I* as a function of *α*. The disease frequency monotonically decreases with *α*, reflecting the pattern observed in Figure 7a that *α* speeds up the time at which disease becomes rare. In contrast to the dramatic effect the assortative meeting parameter *α* has on disease frequency in the short term (Figure 7a), its long-term effect is negligible (Figure 7b).

## Discussion

We have studied a model of the joint dynamics of anti-vaccine sentiment and a vaccine-preventable disease, modelling the sentiment as a contagion itself: a cultural pathogen. We found that the disease and sentiment contagions have closely linked dynamics. The transient dynamics of anti-vaccine sentiment can cause repeated, easily triggered epidemics after long periods of disease absence. Presence of anti-vaccine sentiment and that of the disease are closely coupled: in general, at equilibrium, both are expected in the population or neither.

### Consequences for anti-vaccine sentiment

We find that unless the disease would have been endemic in the absence of anti-vaccine sentiment, disease can always be eliminated at equilibrium through changes only in sentiment parameters. The DSFE appears in Figure 2a–c, and in most situations, a decrease in the sentiment transmission rate *c* or an increase in the pro-vaccine decision rate *w* enables the population to reach the DSFE and to eliminate disease. These results highlight the value of influencing sentiment behaviour as a management strategy for controlling disease.

Our analyses of specific disease parameters (Figure 3) revealed a difference between polio and measles owing to the greater *R*_0_ for measles. In particular, we found that for diseases such as measles with high *R*_0_, endemism of the disease is tightly coupled to endemism of anti-vaccine sentiment, so that the disease is only present if sentiment is present. For diseases with high *R*_0_, it is thus particularly important to maintain low *C*_0_ to avoid epidemics. The more transmissible the disease, the more important it is to decrease sentiment transmission.

The oscillations in Figures 4–6 demonstrate the power of sentiment dynamics to change transient disease dynamics. In Figure 5, sentiment dynamics force the system out of a sequence of oscillations or a steady approach to the DFE or EE, increasing disease frequency via sentiment spread.

Assortative meeting both intensifies epidemics and damps the spread of sentiment and disease. Under high assortativity, disease susceptibility is disproportionately found among anti-vaccine individuals. Individuals therefore only have a risk of becoming disease-infected if they have prolonged disease susceptibility owing to anti-vaccine sentiment; if undecided individuals do convert to the anti-vaccine class, then their risk of getting infected with the disease is dramatically increased.

The concentration of disease in the anti-vaccine class is not unlike scenarios with spatially localized disease reservoirs, such as nosocomial drug-resistant infections (e.g. Lipsitch, Bergstrom, & Levin, 2000). Hospitalization is analogous to membership in the anti-vaccine class; similarly to the spread of disease in the anti-vaccine class by assortative meeting, assortative meeting of hospitalized individuals spreads drug-resistant strains of an infection within the hospital. In the non-hospitalized class, both susceptibility, via general overall health, and exposure, through assortative meeting with contacts who are generally non-hospitalized, are lower, so that disease remains largely localized.

### Broadening the assumptions about transmission

Disease-behaviour analyses often examine highly structured populations in the form of networks. Our model instead focuses on a well-mixed population. This approach gives an analytically tractable model that can formalize qualitative results obtained from less tractable network models (e.g. Salathé & Bonhoeffer, 2008). A well-mixed model also provides a baseline against which to compare more complicated structured population models. In our case, we were able to examine assortative meeting by sentiment type with a minor modification; other violations of the well-mixed-population assumption could also be analysed.

Our model fixes parameters for anti-vaccine sentiment transmission, examining the ensuing dynamics. Other studies have considered different aspects of vaccination behaviour. For example, game-theoretic studies that have modelled vaccine decision-making (e.g. Bauch & Earn, 2004; Fu, Rosenbloom, Wang, & Nowak, 2010) generally conclude that it is difficult to eliminate a disease from the population; when disease prevalence is low, reduced individual incentive to vaccinate increases disease prevalence. A second class of studies has modelled traits such as fear of a disease (e.g. Perra et al., 2011) that induce transmission-reducing behaviours. These studies also find a relaxation of behavioural changes under low disease prevalence, leading to difficulties in long-term management of the disease. Although we did not include lethality from disease or a behavioural response to disease severity, our model accords with these previous results in finding that disease elimination is tied to disease-related behaviour – the elimination of sentiment. Even without feedback from disease prevalence to behaviour in the form of a direct impact on sentiment parameters, we still observe behaviourally driven disease rescue when the time-scale of sentiment transmission acts as an effective relaxation of disease-preventative behaviours (Figure 5). Our model therefore suggests that significant effects of anti-vaccine sentiment on disease dynamics can occur without complex mechanisms of sentiment uptake or transmission.

We did not consider a number of phenomena potentially relevant to sentiment transmission dynamics. Our transmission mechanism was constructed to be simple, with the cultural pathogen transmitted by contact in a well-mixed population. However, social contagions might require multiple interactions before transmission (e.g. Centola, 2010), so that transmission mechanisms are not well approximated by an assumption of a single contact. Thus, for example, Pires and Crokidakis (2017) adopted a majority-rule transmission mechanism in groups of three contacts. With multiple required interactions, Campbell and Salathé (2013) found that anti-vaccine sentiment can spread more slowly, but that the outbreaks can be more intense owing to the increased positive feedback tendencies of the sentiment spread. This and another study (Salathé & Bonhoeffer, 2008) allowed opinion formation on a contact network, and found that increased opinion clustering dramatically increased the probability of an epidemic when the population was close to the minimal susceptible threshold needed to prevent outbreaks. Anti-vaccine sentiment can also be transmitted through centralized sources, such as governmental or media sources, with strong effects of such transmission on maintenance of deleterious cultural traits (Funk et al., 2010; Yeaman, Schick, & Lehmann, 2012); government or media transmission could be treated as a basis for spontaneous acquisition of anti-vaccine sentiment. Thus, the sentiment-only model of Hill, Rand, Nowak, and Christakis (2010) incorporates both transmissible sentiment and sentiment acquired by individual decision-making via spontaneous conversion. Permitting more sentiment compartments or using more complex contagion dynamics would extend the approach to cases with more detailed knowledge available about sentiment behaviours. A feedback of disease prevalence to sentiment dynamics (Goldstein, Philipson, Joo, & Daum, 1996; Bauch & Earn, 2004) would entail changing the constant sentiment transmission and pro-vaccine decision rates into decreasing and increasing functions of disease prevalence, respectively. This modification has the potential to create cyclic behaviour that is not observed in our current model.

Further modelling of sentiment transmission could also make use of mechanisms for social learning uncovered by the study of cultural transmission (e.g. Laland, 2004; Mesoudi & Whiten, 2008; Rendell et al., 2011; Hoppitt & Laland, 2013; Mesoudi, 2016). Beyond the simple ‘unbiased’ transmission (e.g. Cavalli-Sforza & Feldman, 1981; Neiman, 1995) that we use, such mechanisms include conformity bias towards high-prevalence traits (e.g. Henrich & Boyd, 1998; Reader, Bruce, & Rebers, 2008; Muthukrishna, Morgan & Henrich, 2016), payoff bias towards traits that appear to be better or that are exhibited by successful individuals (e.g. Boyd & Richerson, 1985; Mesoudi, 2008; Tanaka et al., 2009; Henrich & Henrich, 2010; Molleman, van den Berg, & Weissing, 2014), and a mixture of social learning strategies in a heterogenous population (e.g. McElreath et al., 2005; Efferson, Lalive, Richerson, McElreath, & Lubell, 2008).

### Additional limitations and extensions

Other potential extensions include addition of age structure and the possibility of opposing social contagions. Age structure is important, as parents make vaccination decisions for children; parental decisions could be included by allowing new births to enter the population as anti-vaccine instead of undecided, at rates proportional to the prevalence of anti-vaccine sentiment among parents. Other ways of including parental decisions include a two-class population modelling approach with parents and children (Eames, 2009) or a general age-structured co-infection approach (Castillo-Chavez, Hethcote, Andreasen, Levin, & Liu, 1989).

Our model does not allow pro-vaccine individuals to transition to the undecided or anti-vaccine classes. This choice allowed us to interpret pro-vaccine sentiment as conferring immunity to anti-vaccine sentiment, enabling a perspective of a cultural inoculation against anti-vaccine sentiment (McGuire & Papageorgis, 1961; Banas & Rains, 2010). A model that permits the undecided class to be replenished by a conversion rate from the pro-vaccine class would be analogous to a SIRS model rather than an SIR model, as the class susceptible to the cultural pathogen can be re-entered (Hethcote, 2000); this change could generate further oscillatory behaviour.

We also do not provide different rates for sentiment switching based on the disease state of an individual. For instance, anti-vaccine individuals who have been infected by the disease have the same rate of becoming pro-vaccine as those who have not been infected. In practice, sentiment switching could depend on disease state, with, for example, a higher sentiment-switching rate in the disease-infected class. However, adoption of vaccine-refusal behaviours is complex, potentially arising from reasons that are not related to the disease itself or leading to a preference for acquiring the disease rather than getting vaccinated (Dubé et al., 2015; Reich, 2016), so that sentiment-switching might have a complex relationship with disease state. A more general model that examines the relationship of vaccine refusal behaviours to disease state could potentially relax the assumption.

Our model also does not treat pro-vaccine sentiment as a contagion. This choice follows empirical evidence (Salathé et al., 2013), but does not preclude incorporation of a transmissible manner of expressing pro-vaccine sentiment. Understanding competing effects of transmissible pro-vaccine and anti-vaccine sentiment could facilitate analysis of the effects of a public policy that uses transmissible pro-vaccine sentiment as a management strategy.

For empirical application of our model, it would be possible to fit the sentiment model to observed trajectories of anti-vaccine sentiment prevalence and to then explore the effect that these dynamics would have on the corresponding disease, or perhaps on other diseases if anti-vaccine sentiment is correlated across diseases. Model-fitting to such data has been performed by Bauch and Bhattacharyya (2012), and sentiment trajectories could be obtained using social media in the manner of studies of Salathé and Khandelwal (2011) and Salathé et al. (2013).

## Conclusions

Early mathematical models of cultural evolution recognized the connection between cultural evolution and disease-transmission models, such as in analogies to susceptible, infected and resistant states for being unaware of a cultural trait, aware of the trait and having adopted the trait (Cavalli-Sforza & Feldman, 1981). We find that using a direct analogy between a biological pathogen and a cultural pathogen generates an informative interaction between the two pathogens that in turn reveals distinctive dynamics in both the short term and the long term.

The formulation of the anti-vaccine sentiment transmission model as a cultural pathogen in an SIR framework identifies three quantities that affect sentiment dynamics and ultimately disease dynamics: the sentiment transmission rate *c* at which anti-vaccine sentiment is transmitted from the anti-vaccine class to the undecided class, the sentiment switching rate *s* at which the anti-vaccine class converts to pro-vaccine, and the pro-vaccine decision rate *w* at which the undecided class converts to pro-vaccine. Interventions to increase vaccination can potentially target any of these three types of transition – decreasing sentiment transmission to undecided individuals, increasing pro-vaccine decisions among undecided individuals, or increasing sentiment switching among anti-vaccine individuals. The influence of an intervention that produces a change of a given magnitude in one or more of these parameters can potentially be investigated using the model.

Finally, beyond our model, a cultural evolution approach can potentially facilitate examination of other questions raised by the existence of culturally pathogenic anti-vaccine sentiment. An analogy might exist with the virulence–transmissibility tradeoff in biological pathogens (Anderson & May, 1982; May & Anderson, 1983), by which high-virulence pathogens can evolve to be less transmissible than low-virulence pathogens. Such topics could be studied by permitting virulence and transmissibility of anti-vaccine sentiment to be influenced by virulence and transmissibility of the associated disease and studying coevolutionary dynamics over an evolutionary time scale.

## Data Availability

All code used in the numerical analysis is available upon request.

## Supplementary material

For supplementary material accompanying this paper visit http://dx.doi.org/10.1017/ehs.2020.17. click here to view supplementary material
