# Supplementary material for: Modelling anti-vaccine sentiment as a cultural pathogen
Source: Evol Hum Sci. 2020 May 7;2:e21. doi: 10.1017/ehs.2020.17 (PMC10427458; doi:10.1017/ehs.2020.17)
Supplement: Supplementary file 1 [file S2513843X20000171sup001.pdf]

Supplementary online material

Appendix A. Finding the equilibria

To find the equilibria of eq. 2 we set the left-hand-sides of eq. 2 to zero and solve for nine variables. This procedure begins by considering marginal variables for the disease and sentiment subsystems (eq. 3).

Subsystems. Summing over the disease variables to write eq. 2 in terms of U, A, and P yields the following SIR model that is equivalent to eq. 1:

$$\begin{aligned} U' &= b - cAU - (w+b)U \\ A' &= cAU - (s+b)A \\ P' &= sA - bP. \end{aligned} \tag{18}$$

Setting the left-hand sides of eq. 18 to zero yields two choices for the equilibrium value of A: either $\widehat{A} = 0$, in which case $\widehat{U} = b/(w+b)$, or $\widehat{A} > 0$, in which case $\widehat{U} = (s+b)/c$ and $\widehat{A} = b/(s+b) - (w+b)/c$. These results are summarized in Table 5.

Summing over the sentiment variables to write eq. 2 in terms of S, I, and R yields the following equations:

$$\begin{aligned} S' &= b - rIS - (vp+b)S \\ I' &= rIS - (g+b)I \\ R' &= gI - bR, \end{aligned} \tag{19}$$

where $p = (SP)/S$. Setting the left-hand sides of eq. 19 to zero yields two choices for the equilibrium value of I: either $\widehat{I} = 0$, in which case $\widehat{S} = 1 - v(\widehat{SP})/b$, or $\widehat{I} > 0$, in which case $\widehat{S} = (g+b)/r$ and $\widehat{I} = [b - v(\widehat{SP})]/(g+b) - b/r$. These results are also summarized in Table 5.

To recover a standard SIR model (eq. 1) from the disease subsystem (eq. 19), we need $p \approx 1$, so almost all disease-susceptible individuals must be pro-vaccine. We would therefore require that the undecided SU state has a short residence time and that the pro-vaccine decision rate is much faster than the anti-vaccine transmission. Informally, this scenario requires a large pro-vaccine decision rate w that greatly exceeds the anti-vaccine transmission rate c.

DSFE and DFE. Each of the four choices for the pair $(\widehat{A}, \widehat{I})$ leads to one of the four possible equilibria. Setting the left-hand-sides of eq. 2 to zero and solving for all nine variables, making use of the results in Table 5, yields the equilibria.

For the DSFE (eq. 6) and the DFE (eq. 8), the zero values for $\widehat{I}$ and $\widehat{A}$ (Table 5) lead to many of the variables being zero at equilibrium, with the rest straightforward to obtain. Finding the SFE and EE requires more work, as described in the following sections.

Equilibrium	$\widehat{S}$	$\widehat{I}$	$\widehat{R}$	$\widehat{U}$	$\widehat{A}$	$\widehat{P}$
DSFE	$1 - \frac{vw}{(v+b)(w+b)}$	0	$\frac{vw}{(v+b)(w+b)}$	$\frac{b}{w+b}$	0	$\frac{w}{w+b}$
DFE	$1 - \frac{v}{v+b}\left[\frac{s}{c}(C_0-1) + \frac{w}{c}\right]$	0	$\frac{v}{v+b}\left[\frac{s}{c}(C_0-1) + \frac{w}{c}\right]$	$\frac{s+b}{c}$	$\frac{b}{s+b} - \frac{w+b}{c}$	$\frac{s}{c}(C_0-1) + \frac{w}{c}$
SFE	$\frac{g+b}{r}$	$\frac{b - v\widehat{SP}}{g+b} - \frac{b}{r}$	$1 - \frac{g}{r} - \frac{b - v\widehat{SP}}{g+b}$	$\frac{b}{w+b}$	0	$\frac{w}{w+b}$
EE	$\frac{g+b}{r}$	$\frac{b - v\widehat{SP}}{g+b} - \frac{b}{r}$	$1 - \frac{g}{r} - \frac{b - v\widehat{SP}}{g+b}$	$\frac{s+b}{c}$	$\frac{b}{s+b} - \frac{w+b}{c}$	$\frac{s}{c}(C_0-1) + \frac{w}{c}$

Table 5. Equilibrium values of marginal compartment variables from eq. 2. For the SFE, $\widehat{SP} = [bR_0 + v + w - \sqrt{(bR_0+v+w)^2 - 4vw}]/(2R_0 v)$ (eq. 10). For the EE, $\widehat{SP}$ is obtained from eq. 11.

SFE. For the SFE (eq. 10), the zero value of $\widehat{A}$ (Table 5) means that $\widehat{SA} = 0$. Thus, substituting the result for $\widehat{I}$ in Table 5 into the third equation of eq. 2 yields $\widehat{SU}$ in terms of $\widehat{SP}$. Inserting this result into the first equation of eq. 2 yields a cubic polynomial in $\widehat{SP}$:

$$0 = v^2 R_0^2 (\widehat{SP})^3 - \left[2vbR_0^2 + v(w+v)R_0\right](\widehat{SP})^2 + \left[b^2R_0^2 + b(w+v)R_0 + wv\right](\widehat{SP}) - wb. \tag{20}$$

The polynomial in eq. 20 has three roots. One root is b/v. If $\widehat{SP} = b/v$, then $\widehat{I} \leqslant 0$ (see Table 5), which is impossible given that the SFE requires $\widehat{I} > 0$. The other two roots are:

$$\widehat{SP} = \frac{(bR_0 + v + w) \pm \sqrt{(bR_0 + v + w)^2 - 4vw}}{2R_0 v}. \tag{21}$$

Note that the quantity $b^2R_0^2 + 2bR_0(v+w) + (v-w)^2$ under the radical is always nonnegative.

The condition $\widehat{I} > 0$ determines which root, positive or negative for the sign of the radical, is the equilibrium solution. By substituting the positive root of eq. 21 for $\widehat{SP}$ into $\widehat{I} = [b - v(\widehat{SP})]/(g+b) - b/r$ (Table 5), we obtain $\widehat{I} > 0 \iff \sqrt{(bR_0+v+w)^2 - 4vw} < bR_0 - (2b+v+w)$. If the right-hand side of this expression is negative, then it is not possible to satisfy this condition. For the right-hand side to be positive, we require $R_0 > (2b+v+w)/b$. If the right-hand side is positive, then we can square both sides to obtain

$$\widehat{I} > 0 \iff \frac{2b+v+w}{b} < R_0 < 1 + \frac{vw}{b(b+v+w)}. \tag{22}$$

The right-hand condition of eq. 22 simplifies to $(b+v+w)^2 < vw$, which is impossible for nonnegative values of the parameters. Hence, the positive root in eq 21 is never the SFE equilibrium value of SP. The negative root of eq. 21 must therefore be the SFE equilibrium value of SP whenever such an equilibrium exists.

Substituting the negative root of eq. 21 for $\widehat{SP}$ into $\widehat{I} = [b - v(\widehat{SP})]/(g+b) - b/r$ (Table 5) yields $\widehat{I} > 0 \iff \sqrt{(bR_0+v+w)^2 - 4vw} > 2b+v+w-bR_0$. If the right-hand side of this expression is negative, then the condition is always satisfied. For the right-hand side to be negative, we require $R_0 > (2b+v+w)/b$. If the right-hand side is positive, then we can square both sides to obtain

$$\widehat{I} > 0 \iff R_0 > \frac{1}{1 - \frac{vw}{(v+b)(w+b)}}. \tag{23}$$

It can be shown that $R_0 > (2b+v+w)/b$ implies the right-hand condition of eq. 23, so the condition in eq. 23 is necessary and sufficient for the existence of the SFE.

EE. The EE (eq. 12) can be obtained by first solving for $x = r\widehat{I}$, setting the left-hand sides of eq. 2 to zero. Substituting the result for $\widehat{A}$ from Table 5 into the first equation of eq. 2 yields $\widehat{SU}$ in terms of x. Substituting this result into the second equation of eq. 2 yields $\widehat{SI}$ in terms of x. Substituting both results into the third equation of eq. 2, writing $\widehat{SP}$ in terms of x using the result for $\widehat{I}$ in Table 5, yields

$$0 = E(x) = (x+s+b)(x+v+b)(x+bC_0)\left[\frac{b}{v}\left(1 - \frac{1}{R_0}\right) - \frac{x}{vR_0}\right] - wbx - b^2\left[w + s(C_0 - 1)\right]. \tag{24}$$

Polynomial $E(x)$ (eq. 24) can be written $E(x) = Q(x) - L(x)$, where $Q(x)$ is a factorizable quartic polynomial and $L(x)$ is a line:

$$Q(x) = (x+s+b)(x+v+b)(x+bC_0)\left[\frac{b}{v}\left(1 - \frac{1}{R_0}\right) - \frac{x}{vR_0}\right] \tag{25}$$

$$L(x) = wbx + b^2\left[w + s\left(C_0 - 1\right)\right]. \tag{26}$$

A positive real root x^* of $E(x)$ yields the desired equilibrium quantity $r\widehat{I}$. Once $r\widehat{I}$ is obtained, the other equilibrium values follow. Roots of $E(x)$ are intersections between $Q(x)$ and $L(x)$, points x^* where $Q(x^*) = L(x^*)$. The following argument proves that there is at most one positive real root x^* of $E(x)$, and obtains the existence condition for this root.

We can assume $C_0 > 1$ because we are at the EE, so that $C_0 = 1/\widehat{U} > 1$ (Table 5). We can also assume $R_0 > 1$ because $R_0 = 1/\widehat{S} > 1$ (Table 5). Then $Q(0) = bC_0(s+b)(v+b)(b/v)(1-1/R_0) > 0$, so $Q(x)$ is downward-pointing and has three negative real roots ($-s-b$, $-v-b$, and $-bC_0$) and one positive real root, $b\,(R_0 - 1)$. We can see that $Q(-b) = L(-b) = b^2 s(C_0 - 1)$, so $Q(x)$ and $L(x)$ intersect at $-b$.

Note that all three negative roots of $Q(x)$ are less than $-b$. Local optima of $Q(x)$ are at roots of $Q'(x)$, which is a cubic equation; there can therefore be at most three local optima. There must be at least one local optimum between two nondegenerate real roots. $Q(x)$ has four real roots, so there must be at least three local optima. There are therefore exactly three local optima, only one of which can possibly be located at an x-value greater than $-b$. This optimum must be a maximum, as $Q(x)$ is downward-pointing and the positive real root is unique. As x increases from $-b$, $Q(x)$ must therefore either monotonically decrease (the rightmost local maximum occurs at $x \leqslant -b$) or increase then decrease (the rightmost local maximum occurs for $x > -b$). Because $Q(-b) = L(-b)$, the former case yields no intersection for $x > -b$, and the latter case yields exactly one such intersection. $Q(x)$ and $L(x)$ therefore have at most one positive intersection.

For this intersection to be positive, $Q(x)$ must stay above $L(x)$ until after $x = 0$. Thus, for the positive intersection x^* between $Q(x)$ and $L(x)$ to exist, and therefore the positive real root of $E(x)$ and an endemic equilibrium to exist, we require $Q(0) > L(0)$, which is equivalent to

$$R_0 > \frac{1}{1 - \frac{v}{v+b}\left(\frac{s}{s+b} + \frac{w-s}{c}\right)}. \tag{27}$$

It is possible to write down the positive real root of $E(x)$, but it is unwieldy:

$$x^* = -\frac{1}{3}[b(2-R_0+C_0)+s+v] - \frac{2^{\frac{4}{3}}\{b^2[(C_0+R_0-1)(C_0+R_0) - C_0(R_0+1)+1] - R_0 + R_0^2\} + (s-v)^2 + sv + b(1+R_0-C_0)(s+v)}{\left(18 - 2^{\frac{2}{3}}\right)\left(W + \sqrt{X^2 - 4Y^3}\right)^{\frac{1}{3}}}, \tag{28}$$

where

$$\begin{aligned}
W &= b^3[-2R_0^3 + 3(1-C_0)R_0^2 + 3(1-2C_0+C_0^2) - 2(1-C_0)^3] \\
&\quad - 3b^2[(s+v)((C_0^2 + 4C_0R_0 + R_0^2 - 4R_0 - 2C_0 + 1)] \\
&\quad - 3b[(C_0 - R_0 - 1)(s^2 - 4sv + v^2) - 9vwR_0] + 2(s^2 - v^2)(s-v) - sv(s+v) \\
X &= -2b^3R_0^3 - 3b^2R_0^2[b(C_0-1) + s + v] + 3bR_0[b^2(C_0-1)^2 - 4b(s+v)(C_0-1) + s^2 - 4sv + v^2 + 27bvw] \\
&\quad + 2b^3(C_0^3 - 1) - 3b^2C_0^2(2b+s+v) + 3bC_0[2b(b+s+v) - (s^2 - 4sv + v^2)] \\
&\quad - 3b^2(s+v) + 3b(s^2 - 4sv + v^2) + 2(s^2 - v^2)(s-v) - sv(s+v) \\
Y &= b^2[c_0^2 - C_0 + 1 + (C_0 + R_0)(R_0 - 1)] + s^2 - sv + v^2 - b(s+v)(C_0 - R_0 - 1).
\end{aligned}$$

Appendix B. Stability conditions for the DSFE

We use a linear stability analysis to determine the stability conditions for the four equilibria. An equilibrium is stable via this analysis if the eigenvalues of the Jacobian matrix evaluated at that equilibrium all have negative real part (Guckenheimer and Holmes, 1990). As the nine variables in eq. 2 sum to 1, we can drop one of them; we drop RP. Element (i,j) of the 8×8 Jacobian matrix J of the system in eq. 2—without RP—is computed by taking the derivative of the ith equation in eq. 2 with respect to the variable in the jth equation (Guckenheimer and Holmes, 1990). Note that in eq. 2, we use $I = IU + IA + IP$ and $A = SA + IA + RA$;

to compute the Jacobian matrix, we must first replace I and A in eq. 2 with these expressions.

$$J = \begin{bmatrix} -rI-cA-w-b & -c(SU) & 0 & -r(SU) & -(r+c)(SU) & -r(SU) & 0 & -c(SU) \\ cA & c(SU)-rI-s-b & 0 & -r(SA) & c(SU)-r(SA) & -r(SA) & 0 & c(SU) \\ w & s & -rI-v-b & -r(SP) & -r(SP) & -r(SP) & 0 & 0 \\ rI & -c(IU) & 0 & r(SU)-cA-g-w-b & r(SU)-c(IU) & r(SU) & 0 & -c(IU) \\ 0 & rI+c(IU) & 0 & r(SA)+cA & r(SA)+c(IU)-g-s-b & r(SA) & 0 & c(IU) \\ 0 & 0 & rI & r(SP)+w & r(SP)+s & r(SP)-g-b & 0 & 0 \\ 0 & -c(RU) & 0 & g & -c(RU) & 0 & -cA-w-b & -c(RU) \\ 0 & c(RU) & 0 & 0 & c(RU)+g & 0 & cA & c(RU)-s-b \end{bmatrix}. \tag{29}$$

For the DSFE, we have the following eigenvalues:

$$\begin{aligned} \lambda_1 &= -s-b-g < 0 \\ \lambda_2 &= -w-b-g < 0 \\ \lambda_3 &= -s-b < 0 \\ \lambda_4 &= \frac{bc}{w+b} - s - b \\ \lambda_5 &= \frac{rb}{w+b}\left(1 + \frac{w}{v+b}\right) - g - b \\ \lambda_6 &= -w-b < 0 \\ \lambda_7 &= -w-b < 0 \\ \lambda_8 &= -v-b < 0. \end{aligned}$$

The conditions for stability of the DSFE are therefore

$$\lambda_4 < 0 \iff C_0 < 1 + \frac{w}{b} \tag{30}$$

$$\lambda_5 < 0 \iff R_0 < \frac{1}{1 - \frac{vw}{(v+b)(w+b)}}. \tag{31}$$

Appendix C. Stability conditions for the DFE

Using the Jacobian matrix (eq. 29), we have the following eigenvalues for the DFE:

$$\begin{aligned} \lambda_1 &= -bC_0 < 0 \\ \lambda_2 &= -s-b < 0 \\ \lambda_3 &= -s-b-g < 0 \\ \lambda_4 &= -bC_0 - g < 0 \\ \lambda_5 &= r\left[1 - \frac{v}{v+b}\left(\frac{s}{s+b} + \frac{w-s}{c}\right)\right] - b - g \\ \lambda_6 &= -b-v < 0 \\ \lambda_7 &= \frac{1}{2}\left[-bC_0 + \sqrt{(bC_0)^2 + 4(s+b)\left[(w+b) - bC_0\right)]}\right] \\ \lambda_8 &= \frac{1}{2}\left[-bC_0 - \sqrt{(bC_0)^2 + 4(s+b)\left[(w+b) - bC_0\right)]}\right] < 0. \end{aligned}$$

The conditions for stability of the DFE are therefore

$$\lambda_5 < 0 \iff R_0 < \frac{1}{1 - \frac{v}{v+b}\left(\frac{s}{s+b} + \frac{w-s}{c}\right)} \tag{32}$$

$$\lambda_7 < 0 \iff C_0 > 1 + \frac{w}{b}. \tag{33}$$

Appendix D. Stability conditions for the SFE

Using the Jacobian matrix (eq. 29), we have the following eigenvalues for the SFE:

$$\begin{aligned}\lambda_1 &= -s - b - g < 0\\ \lambda_2 &= -s - r\widehat{I} - b < 0\\ \lambda_3 &= \frac{bc}{w+b} - s - b\\ \lambda_4 &= -w - b < 0\\ \lambda_5 &= -w - b - g < 0.\end{aligned}$$

The remaining eigenvalues are the roots of the following cubic equation:

$$x^3 + \Big[2(r\widehat{I}+b)+v+w\Big]x^2 + \Big[(r\widehat{I})^2 + r\widehat{I}(3b+g+v+w)+(v+b)(w+b)\Big]x + r\widehat{I}(g+b)[2(r\widehat{I}+b)+v+w-bR_0]. \tag{34}$$

We can show that the roots of eq. 34 all have negative real part—and that therefore none of these eigenvalues affect the stability of the SFE—by using the Routh-Hurwitz criterion concerning the signs of coefficients of the polynomial and the signs of certain functions of the coefficients (Gantmacher, 1960; Meinsma, 1995).

For the polynomial in eq. 34 to satisfy the Routh-Hurwitz criterion, and to therefore conclude that all its roots have negative real part, we require first that all coefficients of the polynomial have the same sign. Label the coefficient of x^i in eq. 34 b_i. Then we have:

$$\begin{aligned}b_0 &= r\widehat{I}(g+b)[2(r\widehat{I}+b)+v+w-bR_0]\\ b_1 &= (r\widehat{I})^2 + r\widehat{I}(3b+g+v+w)+(v+b)(w+b)\\ b_2 &= 2(r\widehat{I}+b)+v+w\\ b_3 &= 1.\end{aligned} \tag{35}$$

For $i = 1,2,3$, $b_i > 0$, as sums of positive quantities (eq. 35). For b_0, note that at the SFE, $\widehat{I} = [b - v(\widehat{SP})]/(g+b) - b/r > 0$ (Table 5), so $r\widehat{I} = bR_0 - vR_0(\widehat{SP}) - b$ by eq. 4. Because $0 \leqslant \widehat{SP} \leqslant \widehat{S} = 1/R_0$ (Table 5), we have the following bounds on $r\widehat{I}$:

$$b(R_0-1) - v \leqslant r\widehat{I} \leqslant b(R_0-1). \tag{36}$$

The condition $b_0 > 0$ is equivalent to $r\widehat{I} > \frac{1}{2}(bR_0 - v - w - 2b)$. From eq. 36, $r\widehat{I} \geqslant bR_0 - b - v > bR_0 - v - w - 2b > \frac{1}{2}(bR_0 - v - w - 2b)$. Consequently, $b_0 > 0$, so all coefficients of eq. 34 are positive.

For the second part of the Routh-Hurwitz criterion, we must also show that several terms computed from these coefficients are positive. A cubic polynomial has only one additional condition that must be shown: $b_2b_1 - b_3b_0 > 0$. We have

$$\begin{aligned}b_2b_1 - b_3b_0 &= b_2b_1 - r\widehat{I}(g+b)(b_2 - bR_0)\\ &= b_2\Big[(r\widehat{I})^2 + r\widehat{I}(3b+g+v+w)+(v+b)(w+b) - r\widehat{I}(g+b)\Big] + r\widehat{I}(g+b)bR_0\\ &= b_2\Big[(r\widehat{I})^2 + r\widehat{I}(2b+v+w)+(v+b)(w+b)\Big] + r\widehat{I}(g+b)bR_0 > 0.\end{aligned}$$

We conclude that by the Routh-Hurwitz criterion, all roots of the polynomial in eq. 34—which are eigenvalues λ_6, λ_7, and λ_8—have negative real part. Hence, provided the SFE exists (i.e. the condition in eq. 23 is satisfied), the stability condition for the SFE is:

$$\lambda_3 < 0 \iff C_0 < 1 + \frac{w}{b}.$$

Appendix E. Stability conditions for the EE

Using the Jacobian matrix (eq. 29), we have the following eigenvalues for the EE:

$$\begin{aligned}
\lambda_1 &= -bC_0 - g < 0 \\
\lambda_2 &= -s - b - g < 0 \\
\lambda_3 &= \frac{1}{2}\left[-bC_0 - \sqrt{(bC_0)^2 + 4(s+b)(w+b-bC_0)}\right] < 0 \\
\lambda_4 &= \frac{1}{2}\left[-bC_0 + \sqrt{(bC_0)^2 + 4(s+b)(w+b-bC_0)}\right].
\end{aligned}$$

The remaining eigenvalues are the roots of a quartic equation:

$$\begin{aligned}
&x^4 + \left[3r\widehat{I} + b(2+C_0) + s + v\right] x^3 \\
&+ \left[3(r\widehat{I})^2 + r\widehat{I}\,[b(5+2C_0) + g + 2(s+v)] + b^2(1+2C_0) + sv + b(1+C_0)(s+v)\right] x^2 \\
&+ \left[(r\widehat{I}+s+b)(r\widehat{I}+v+b)(r\widehat{I}+bC_0) + (g+b)\left[3(r\widehat{I})^2 - (bR_0 - bC_0 - 2b - s - v)(r\widehat{I})\right]\right] x \\
&+ (g+b)r\widehat{I}\left[(r\widehat{I}+bC_0)(r\widehat{I}+b+s+v) + sv + (r\widehat{I}+b-bR_0)(2r\widehat{I}+v+b+s+bC_0)\right] = 0.
\end{aligned} \tag{37}$$

One condition required for stability of the EE is:

$$\lambda_4 < 0 \iff C_0 > 1 + \frac{w}{b}. \tag{38}$$

As we did with the SFE, we use the Routh-Hurwitz criterion to show that the roots of eq. 37 all have negative real part—and that therefore none of these eigenvalues affect the stability of the EE. Eq. 37 has coefficients

$$\begin{aligned}
b_0 &= (g+b)r\widehat{I}\left[(r\widehat{I}+bC_0)(r\widehat{I}+b+s+v) + sv + (r\widehat{I}+b-bR_0)(2r\widehat{I}+v+b+s+bC_0)\right] \\
b_1 &= (r\widehat{I}+s+b)(r\widehat{I}+v+b)(r\widehat{I}+bC_0) + (g+b)\left[3(r\widehat{I})^2 - (bR_0 - bC_0 - 2b - s - v)(r\widehat{I})\right] \\
b_2 &= 3(r\widehat{I})^2 + r\widehat{I}\,[b(5+2C_0) + g + 2(s+v)] + b^2(1+2C_0) + sv + b(1+C_0)(s+v) \\
b_3 &= 3r\widehat{I} + b(2+C_0) + s + v \\
b_4 &= 1.
\end{aligned} \tag{39}$$

We see that as sums of positive quantities, $b_i > 0$ for $i = 2, 3, 4$. We must next show $b_0, b_1 > 0$.

Additional bounds on $r\widehat{I}$. To aid in the proof, we derive further bounds on the value of $r\widehat{I}$ for the EE in addition to eq. 36. In particular, we obtain a tighter bound on $r\widehat{I}$ than eq. 36 by noticing that a necessary condition for the EE is $w \leqslant b(C_0 - 1)$ (from the requirement $\lambda_4 < 0$, eq. 38). We can create a new function

$$L_m(x) = b^2(C_0 - 1)(x + s + b),$$

which is greater than or equal to $L(x)$ (eq. 26) if $x \geqslant 0$.

Consider the process we used to study the roots of eq. 24 in Appendix A. Let $E_m(x) = Q(x) - L_m(x)$. Then for some $x_1 > 0$, $E_m(x_1) = 0$ implies $Q(x_1) = L_m(x_1) \geqslant L(x_1)$. From our discussion in Appendix A, we know that for a positive intersection x^* between $Q(x)$ and $L(x)$ to exist, $Q(x) > L(x)$ for $0 \leqslant x < x^*$ and $Q(x) < L(x)$ for $x^* < x$. Thus, $x_1 < x^*$, so a real positive root of $E_m(x)$—in particular, the largest positive real root, when it exists—provides a lower bound on the real positive root of $E(x)$ (eq. 24), which is the equilibrium value of $r\widehat{I}$. Of the four roots of $E_m(x)$, two are always negative ($-b$ and $-s-b$), one is

$$x_1 = \frac{1}{2}\left[bR_0 - bC_0 - b - v + \sqrt{(bR_0 - bC_0 - b - v)(1 + 4bC_0) + 4b^2C_0^2}\right], \tag{40}$$

and the other is the same as that in eq. 40 except for a negative sign in front of the radical term; this root is therefore smaller than x_1 and we do not need to consider it.

$b_1 > 0$ **in eq. 39.** Note that b_1 (eq. 39) is the following cubic polynomial $G(t)$ evaluated at $t = r\widehat{I}$:

$$G(t) = (t+s+b)(t+v+b)(t+bC_0) + (g+b)\left[3t^2 - (bR_0 - bC_0 - 2b - s - v)t\right].$$

This expression consists of the sum of a positive cubic polynomial and an upward-pointing parabola with roots at 0 and $t_1 = \frac{1}{3}(bR_0 - bC_0 - 2b - s - v)$. If $t_1 \leqslant 0$, then $G(t) \geqslant (t+s+b)(t+v+b)(t+bC_0) > 0$ for all $t \geqslant 0$. If $t_1 > 0$, then $G(t) \geqslant (t+s+b)(t+v+b)(t+bC_0) > 0$ for all $t \geqslant t_1$. Thus, $G(t) > 0$ for all $t \geqslant \max(0, t_1)$. Because $r\widehat{I} > 0$—guaranteed by eq. 27—to show that the coefficient b_1 of x in eq. 37 is positive, it suffices to show that $r\widehat{I} > t_1$. The result follows immediately from eq. 36, as $r\widehat{I} \geqslant bR_0 - v - b > bR_0 - bC_0 - 2b - s - v = 3t_1 \geqslant t_1$.

$b_0 > 0$ **in eq. 39.** To evaluate the sign of b_0 (eq. 39) at equilibrium, we consider two quadratic polynomials. First, we examine $q(t)$, the negative of the quadratic polynomial for which the lower bound x_1 (eq. 40) is the larger root (taking the negative does not change the root):

$$q(t) = t^2 + [b(1 + C_0 - R_0) + v]\,t - b^2C_0(R_0 - 1) + vb(C_0 - R_0).$$

The second polynomial, $g(t)$, is equal to the quadratic part of b_0 (eq. 39) when evaluated at $t = r\widehat{I}$:

$$g(t) = 3t^2 + 2\,[b(2 + C_0 - R_0) + s + v]\,t - b^2C_0(R_0 - 1) + b(1 + C_0 - R_0)(s + b + v) + sv. \qquad (41)$$

Because $b_0 = (g+b)r\widehat{I}g(r\widehat{I})$, the signs of $g(t)$ (eq. 41) and b_0 (eq. 39) are the same. We must show $g(t) > 0$.

We first note that $g(t)$ and $q(t)$ are upward-pointing parabolas that intersect at $x_2 = -s - b < 0$ and $2x_3 = bR_0 - bC_0 - b - v$. In addition, $q(0) - g(0) = 2(s+b)x_3$, so x_3 and $q(0) - g(0)$ have the same sign. If $q(0) \geqslant g(0)$, then $x_3 \geqslant 0$. In this case, $q(t) \geqslant g(t)$ at $t = 0$ but then $q(t)$ becomes less than $g(t)$ for $t > x_3$, with no further change in relative size, as no further intersections occur between $g(t)$ and $q(t)$ for $t > x_3$. Crucially, $x_3 < x_1 \leqslant r\widehat{I}$ (eq. 40), so $g(r\widehat{I}) > g(x_1) \geqslant q(x_1) = 0$, which is what we wanted to show.

If instead $g(0) < q(0)$, then both intersections x_2 and x_3 are negative, so the relationship $g(t) > q(t)$ continues to be true for all $t > 0$, and in particular we have $g(t) > g(x_1) \geqslant q(x_1) = 0$. In either case, $g(t) > 0$, and so b_0 (eq. 39) is positive. We can therefore proceed with using the Routh-Hurwitz criterion.

Notation for demonstrating that the additional terms of the Routh-Hurwitz criterion are positive. We define the following always-positive quantities to simplify the notation:

$$\begin{aligned} k_s &= t + s + b \\ k_v &= t + v + b \\ k_c &= t + bC_0. \end{aligned} \qquad (42)$$

Using the quantities in eq. 42, we can write coefficients from eq. 39 used in eqs. 43 and eqs. 44 as

$$\begin{aligned} b_0 &= [(g+b)(k_sk_c + k_vk_c + k_sk_v) - br(k_s + k_v + k_c - x - b)]\,t \\ b_1 &= k_sk_vk_c + [(g+b)(k_s + k_v + k_c) - br]\,t \\ b_2 &= k_sk_c + k_vk_c + k_sk_v + (g+b)t \\ b_3 &= k_s + k_v + k_c \\ b_4 &= 1. \end{aligned}$$

The Routh-Hurwitz criterion for a quartic polynomial requires the following two terms to be positive:

$$b_3b_2 - b_4b_1 > 0 \qquad (43)$$

$$(b_3b_2 - b_4b_1)b_1 - b_3^2b_0 > 0. \qquad (44)$$

Demonstrating that the condition in eq. 43 is satisfied. We can verify eq. 43 directly by noting that $b_3b_2 - b_4b_1$ can be written as the following polynomial $d(t)$ evaluated at $t = r\widehat{I}$:

$$d(t) = (k_s + k_c)(k_v + k_c)(k_s + k_v) + brt.$$

Because k_s, k_v, and k_c are greater than 0 if $t \geqslant 0$, $d(r\widehat{I}) > 0$, so the condition in eq. 43 is satisfied.

Demonstrating that the condition in eq. 44 is satisfied.

Refining the condition in eq. 44. To verify eq. 44, we note that the quantity $(b_3b_2 - b_4b_1)b_1 - b_3^2b_0$ can be written as the following polynomial $p(t)$, evaluated at $t = r\widehat{I}$:

$$p(t) = k_sk_vk_c(k_s + k_v)(k_s + k_c)(k_v + k_c) + tp_1(t) + brt^2\left[(g + b)(k_s + k_v + k_c) - br\right], \tag{45}$$

where

$$\begin{aligned} p_1(t) = {} & br\left[(k_s + k_v + k_c)^2(k_s + k_v + k_c - t - b) - (k_s + k_v)(k_s + k_c)(k_v + k_c) + k_sk_vk_c\right] \\ & + (g + b)\left[(k_s + k_v + k_c)(k_s + k_v)(k_s + k_c)(k_v + k_c) - (k_s + k_v + k_c)^2(k_sk_v + k_sk_c + k_vk_c)\right]. \end{aligned}$$

Our goal is to show that $p(r\widehat{I}) > 0$. By inspection, the first of three terms in $p(t)$ (eq. 45) is always positive. With a reminder that $R_0 = \frac{r}{g+b}$, the third term being positive is equivalent to:

$$\begin{aligned} 0 &< brt^2\left[(g + b)(k_s + k_v + k_c) - br\right] \\ 0 &< (g + b)(k_s + k_v + k_c) - br \\ bR_0 &< k_s + k_v + k_c \\ bR_0 &< 3t + 2b + s + v + bC_0 \\ 3t &> bR_0 - 2b - s - v - bC_0. \end{aligned} \tag{46}$$

From eq. 36, we have that $r\widehat{I} \geqslant bR_0 - v - b > bR_0 - 2b - s - v - bC_0 > \frac{1}{3}\left(bR_0 - 2b - s - v - bC_0\right)$, and the condition in eq. 46 is satisfied at $t = r\widehat{I}$.

The first and third terms of $p(t)$ (eq. 45) are positive at $t = r\widehat{I}$. If we can show that the second term is also positive at $t = r\widehat{I}$, then we have shown that $p(r\widehat{I}) > 0$ and that therefore the condition in eq. 44 is satisfied. The second term is positive if and only if $p_1(t) > 0$ at $t = r\widehat{I}$. Rearranging $p_1(t) > 0$ yields:

$$bR_0\left[(k_s + k_v + k_c)^2(k_s + k_v + k_c - t - b) - (k_s + k_v)(k_s + k_c)(k_v + k_c) + k_sk_vk_c\right] > k_sk_vk_c(k_s + k_v + k_c). \tag{47}$$

We can demonstrate that the refined condition in eq. 47 is satisfied by bounding the left-hand side of eq. 47 from below and demonstrating that this lower bound exceeds the right-hand side. We can bound the left-hand side of eq. 47 from below as follows:

$$\begin{aligned} & bR_0\left[(k_s + k_v + k_c)^2(k_s + k_v + k_c - t - b) - (k_s + k_v)(k_s + k_c)(k_v + k_c) + k_sk_vk_c\right] \\ & > bR_0\left[(k_s + k_v + k_c)^2(k_s + k_v + k_c - t - b) - (k_s + k_v)(k_s + k_c)(k_v + k_c) - k_sk_vk_c\right] \\ & = bR_0\left(k_s + k_v + k_c\right)\left[k_s^2 + k_v^2 + k_c^2 + k_sk_v + k_sk_c + k_vk_c - (k_s + k_v + k_c)(t + b)\right] \\ & = (k_s + k_v + k_c)\left[p_2(t) + k_sk_vk_c\right], \end{aligned} \tag{48}$$

where

$$\begin{aligned} p_2(t) = {} & bR_0\left[3t^2 + 3(s + v + bC_0 + b)t + bC_0(s + v + bC_0 + b) + s^2 + v^2 + sv + b^2 + 2b(s + v)\right] \\ & - (t + s + b)(t + v + b)(t + bC_0). \end{aligned} \tag{49}$$

Using this lower bound from eq. 48, we obtain a new condition that implies eq. 47:

$$\begin{aligned}(k_s+k_v+k_c)\left[p_2(t)+k_sk_vk_c\right] &> k_sk_vk_c(k_s+k_v+k_c)\\ p_2(t)+k_sk_vk_c &> k_sk_vk_c\\ p_2(t) &> 0.\end{aligned} \tag{50}$$

Showing $p_2(t) > 0$ implies eq. 47, the refinement of the condition of eq. 44.

Outlining the demonstration of the refined condition in eq. 50. We will now build up to demonstrating that eq. 50 is true through a series of smaller computations. To show that eq. 50 holds for $t = r\widehat{I}$, it suffices to show first that $p_2(t)$ (eq. 49) has at most one positive real root, and then that eq. 50 holds for $t = 0$ and some value $t_2 \geqslant r\widehat{I}$. If $p_2(t)$ has at most one positive real root, then $p_2(t)$ has at most one sign change for $t > 0$. If eq. 50 holds for $t = 0$ and some value $t_2 \geqslant r\widehat{I}$, then $p_2(t)$ has the same sign throughout $[0, t_2]$, the possible interval in which $t = r\widehat{I}$ resides, which implies that eq. 50 is true for $t = r\widehat{I}$.

$p_2(t)$ (eq. 49) has one positive real root. To show that $p_2(t)$ (eq. 49) has at most one positive real root (in particular exactly one such root), we use Descartes' rule of signs, which states that the upper bound for the number of positive real roots of a polynomial is the number of sign changes between its coefficients from lowest order to highest order.

The coefficients of $p_2(t)$ are

$$\begin{aligned}c_0 &= bR_0\left[bC_0(s+v+bC_0+b)+s^2+v^2+sv+b^2+2b(s+v)\right]-bC_0(s+b)(v+b)\\ c_1 &= 3(b+bC_0+s+v)bR_0-(b^2+2b^2C_0+bs+bC_0s+bv+bC_0v+sv)\\ c_2 &= 3bR_0-b(2+C_0)-s-v\\ c_3 &= -1.\end{aligned}$$

The cubic coefficient c_3 is obviously negative. The quadratic coefficient c_2 is positive if

$$3bR_0 > 2b+bC_0+s+v. \tag{51}$$

The linear coefficient c_1 is positive if

$$\begin{aligned}3bR_0 &> \frac{b^2+2b^2C_0+bs+bC_0s+bv+bC_0v+sv}{b+bC_0+s+v}\\ &= \frac{(b+bC_0+s+v)(2b+bC_0+s+v)-\left[b^2(1+C_0+C_0^2)+s^2+sv+v^2+b(2+C_0)(s+v)\right]}{b+bC_0+s+v}\\ &= (2b+bC_0+s+v)-\frac{b^2(1+C_0+C_0^2)+s^2+sv+v^2+b(2+C_0)(s+v)}{b+bC_0+s+v}.\end{aligned} \tag{52}$$

Notice that eq. 51 implies eq. 52. It is therefore impossible for the quadratic coefficient to be positive but the linear coefficient to be negative. So, for c_1, c_2, and c_3, we either have $-/-/-$, $+/-/-$, or $+/+/-$ as coefficient signs. If we can show that the constant term in $p_2(t)$ is positive, then our possible coefficient signs are $+/-/-/-$, $+/+/-/-$, or $+/+/+/-$, all of which have exactly one sign change. By Descartes' rule of signs, $p_2(t)$ would have at most one positive real root. We now show that c_0, the constant term of $p_2(t)$ (eq. 49) is positive. Note that this is equivalent to applying $t = 0$ to eq. 50, and thus will also demonstrate that eq. 50 holds for $t = 0$. Applying $t = 0$ to eq. 50 yields $p_2(0) > 0$ if

$$bR_0\left[bC_0(s+v+bC_0+b)+s^2+v^2+sv+b^2+2b(s+v)\right] > bC_0(s+b)(v+b). \tag{53}$$

Using the existence condition for the endemic equilibrium $r\widehat{I}$ from eq. 27 yields

$$\begin{aligned}
bR_0 &> \frac{b}{1-\frac{v}{v+b}\left(\frac{s}{s+b}+\frac{w-s}{c}\right)} \\
&> \frac{b}{1-\frac{v}{v+b}\left(\frac{s}{s+b}-\frac{s}{c}\right)} \\
&= \frac{bC_0(s+b)(v+b)}{b^2C_0+sv+bC_0(s+v)} \\
bR_0[bC_0(s+v+b)+sv] &> bC_0(s+b)(v+b).
\end{aligned} \tag{54}$$

The left-hand side of this condition is strictly less than the left-hand side of eq. 53. Hence, the sign of the constant term of $p_2(t)$ is positive, which means that by Descartes's rule of signs, $p_2(t)$ has at most one positive real root. In fact, $p_2(t)$ has exactly one positive real root: its leading coefficient c_3 is negative and it has positive y-intercept $p_2(0) > 0$, so it must have at least one positive real root as well.

Further refining the remaining condition for $p_2(r\widehat{I}) > 0$. In the previous section, we have shown that $p_2(t)$ has one real positive root. In the process, we have also shown that $p_2(0) > 0$. $p_2(t)$ is a downward-pointing cubic polynomial with positive y-intercept and one real positive root, so it must change sign only once on the interval $t \in [0, \infty)$. If we can show that $p_2(t_2) > 0$ for some $t_2 \geqslant r\widehat{I} > 0$, then we know that this sign change must occur for $t > t_2$, and so $p_2(r\widehat{I}) > 0$.

Let $t_2 = bR_0 - b$. We know that $t_2 \geqslant r\widehat{I}$ by eq. 36. Evaluating $p_2(t_2)$ yields:

$$\begin{aligned}
p_2(t_2) &= bR_0\left[3\left(bR_0-b\right)^2+3(s+v+bC_0+b)\left(bR_0-b\right)+bC_0(s+v+bC_0+b)+s^2+v^2+sv+b^2+2b(s+v)\right] \\
&\quad -(bR_0+s)(bR_0+v)(bR_0+bC_0-b) \\
&= bR_0\left[2(bR_0)^2+2\left(b(C_0-1)+s+v\right)bR_0+\left(s^2+v^2+b^2(C_0-1)^2\right)\right]-b(C_0-1)sv \\
&= p_3(bR_0),
\end{aligned}$$

where

$$p_3(t) = t\left[2t^2+2[b(C_0-1)+s+v]t+\left(s^2+v^2+b^2(C_0-1)^2\right)\right]-b(C_0-1)sv.$$

To show $p_2(t_2) > 0$, it suffices to show

$$p_2(t_2) = p_3(bR_0) > 0. \tag{55}$$

To facilitate this proof, we rewrite $p_3(t)$ as follows:

$$p_3(t) = t\left[t^2+bC_0(s+v+b)+sv+T(t)\right]-b(C_0-1)sv,$$

where

$$T(t) = [t+s+v+b(C_0-1)]^2-2[b(C_0-1)(s+v)+sv]-[bC_0(s+v+b)+sv]. \tag{56}$$

Demonstrating that $T(bR_0) > 0$. We can show that $T(bR_0) > 0$ by noting that $T(t)$ (eq. 56) is an upward-pointing quadratic polynomial with larger root

$$t_3 = b-bC_0-s-v+\sqrt{b^2C_0-2bs+3bC_0s-2bv+3bC_0v+3sv}$$

If we can show that $t_3 \leqslant b$, then because $T(t)$ (eq. 56) is upward-pointing and $R_0 > 1$ (eq. 27), then we would have $T(bR_0) > T(b) \geqslant T(t_3) = 0$. $t_3 \leqslant b$ if and only if

$$\begin{aligned}
bC_0+s+v &\geqslant \sqrt{b^2C_0-2bs+3bC_0s-2bv+3bC_0v+3sv} \\
(bC_0+s+v)^2 &\geqslant b^2C_0-2bs+3bC_0s-2bv+3bC_0v+3sv \\
T_1(bC_0) &\geqslant 0,
\end{aligned}$$

where we can square both sides because both sides are positive, and

$$T_1(t) = t^2 - (s+v+b)t + (s-v)^2 + sv + 2b(s+v). \tag{57}$$

The quadratic polynomial $T_1(t)$ (eq. 57) is upward-pointing and has larger root

$$t_4 = \frac{1}{2}\left[s+v+b+\sqrt{(b-s-v)^2 - 4\left[s^2 - sv + v^2 + b(s+v)\right]}\right].$$

If we can show that $t_4 \leqslant b$, then because $T_1(t)$ (eq. 56) is upward-pointing and $C_0 > 1$ (eq. 38), $T_1(bC_0) > T_1(b) \geqslant T_1(t_4) = 0$. $t_4 \leqslant b$ if and only if:

$$\begin{aligned} b &\geqslant \frac{1}{2}\left[s+v+b+\sqrt{(b-s-v)^2 - 4\left[s^2 - sv + v^2 + b(s+v)\right]}\right] \\ b-s-v &\geqslant \sqrt{(b-s-v)^2 - 4\left[s^2 - sv + v^2 + b(s+v)\right]}. \end{aligned}$$

The condition for t_4 to exist is

$$\begin{aligned} (b-s-v)^2 &\geqslant 4\left[s^2 - sv + v^2 + b(s+v)\right] \\ 0 &\leqslant b^2 - 6(s+v)b - 3(s-v)^2. \end{aligned}$$

This condition is an upward-pointing parabola in terms of b with negative y-intercept. The value of b must therefore be greater than the positive real root of this parabola, or

$$\begin{aligned} b &\geqslant 3(s+v) + 2\sqrt{3(s^2+sv+v^2)} \\ b-s-v &\geqslant 2\left[s+v+\sqrt{3(s^2+sv+v^2)}\right] > 0. \end{aligned}$$

Thus, for t_4 to exist, we need $b-s-v > 0$.

Note that if t_4 does not exist, then $T_1(t) > 0$ for all t, and in particular, $T_1(bC_0) > 0$. We therefore have addressed the case where $b-s-v \leqslant 0$ and the case where t_4 does not exist but $b-s-v > 0$. If $b-s-v > 0$ and t_4 exists, then we have:

$$\begin{aligned} (b-s-v)^2 &\geqslant (b-s-v)^2 - 4\left[s^2 - sv + v^2 + b(s+v)\right] \\ (b-s-v)^2 &\geqslant (b-s-v)^2 - 4\left[(s-v)^2 + sv + b(s+v)\right], \end{aligned}$$

which is always true. Hence, $t_4 \leqslant b$ if t_4 exists, so $T_1(bC_0) > 0$. In turn, $t_3 \leqslant b$, and $T(bR_0) > 0$.

Demonstrating $p_2(r\widehat{I}) > 0$ through the refined condition in eq. 55. Using the fact that $T(bR_0) > 0$, we can proceed to demonstrate the condition in eq. 55:

$$\begin{aligned} p_3(bR_0) &= bR_0\left[(bR_0)^2 + bC_0(s+v+b) + sv + T(bR_0)\right] - b(C_0-1)sv \\ &> bR_0\left[(bR_0)^2 + bC_0(s+v+b) + sv\right] - b(C_0-1)sv \\ &> bR_0\left[bC_0(s+v+b) + sv\right] - b(C_0-1)sv \\ &> bC_0(v+b)(s+b) - b(C_0-1)sv \\ &= b\left[bC_0(s+v+b) + sv\right] \\ &> 0, \end{aligned}$$

where the third inequality follows from eq. 54. Thus, eq. 55 is satisfied, which implies that eq. 50 holds at $t = bR_0 - b$. Eq. 50 then holds at $t = r\widehat{I} \leqslant t_2$, implying that all three terms of eq. 45 are positive at $t = r\widehat{I}$, satisfying eq. 44. By the Routh-Hurwitz criterion, all roots of eq. 37—λ_5, λ_6, λ_7, and λ_8—have negative real part. None of these eigenvalues contributes to determining stability of the EE. Provided the EE exists (i.e. the condition in eq. 27 is satisfied), the stability condition for the EE is therefore eq. 38.

Boundary assignments for stability conditions in Table 3. Table 3 summarizes the results from Appendices A-E. The stability conditions for the DSFE come from eq. 30 and eq. 31, for the DFE from

eq. 33 and eq. 32, for the SFE from eq. 23 and eq. D, and for the EE from eq. 27 and eq. 38. As all these inequalities are strict, to complete Table 3, we identify the equilibria that are stable on the boundaries.

From Table 5, we see that if $C_0 = 1 + \frac{w}{b}$, then we have for the DFE and EE:

$$\begin{aligned}\widehat{A} &= \frac{b}{s+b} - \frac{w+b}{c} \\ &= \frac{b}{c}\left[C_0 - \left(1 + \frac{w}{b}\right)\right] \\ &= 0.\end{aligned}$$

We have shown that if equality occurs in the sentiment condition in Table 3, then $\widehat{A} = 0$, so the DFE is equivalent to the DSFE and the EE is equivalent to the SFE. Hence, the equals sign in the sentiment condition is assigned to the DSFE and the SFE.

Similarly, if $R_0 = \frac{1}{1-\left(\frac{v}{v+b}\right)\left(\frac{w}{w+b}\right)}$, then we have

$$bR_0 + v + w = b + v + w + \frac{vw}{b+v+w},$$

and so for the SFE we have:

$$\begin{aligned}\widehat{I} &= \frac{b - v\widehat{SP}}{g+b} - \frac{b}{r} \\ r\widehat{I} &= R_0(b - v\widehat{SP}) - b \\ r\widehat{I} &= R_0\left[b - v\frac{bR_0 + v + w - \sqrt{(bR_0+v+w)^2 - 4vw}}{2R_0 v}\right] - b \\ &= bR_0 - b - \frac{1}{2}\left[bR_0 + v + w - \sqrt{(bR_0+v+w)^2 - 4vw}\right] \\ &= bR_0 - b - \frac{1}{2}\left[b + v + w + \frac{vw}{b+v+w} - \sqrt{\left(b+v+w - \frac{vw}{b+v+w}\right)^2}\right] \\ &= 0.\end{aligned}$$

Finally, for the EE, if $R_0 = \frac{1}{1-\frac{v}{v+b}\left(\frac{s}{s+b}+\frac{w-s}{c}\right)}$, then the only nonnegative root of eq. 24 is $r\widehat{I} = x^* = 0$ (see Appendix A), and $\widehat{I} = 0$.

We have shown that if equality occurs in the disease condition in Table 3, then $\widehat{I} = 0$, so the SFE is equivalent to the DSFE and the EE is equivalent to the DFE. Hence, the equals sign in the disease condition is assigned to the DSFE and the DFE.

Appendix F. Boundary curves for Figure 2

To generate Figure 2, we rearrange the conditions in Table 3 into the form $w = f_i(c)$, where is $f_i(c)$ is some function of c, $i = A$ for the sentiment condition, and $i = I$ for the disease condition. This rearrangement creates boundary curves in the c-w plane, demarcating regions of stability for the equilibria. The sentiment conditions for stability in Table 3 result in a boundary curve that determines endemism of the sentiment, and the disease conditions in Table 3 result in a boundary curve that determines disease endemism.

In plotting the c-w plane, note that we have a choice among three pairs of sentiment parameters: c and w, s and w, and c and s. Only w appears in all conditions in Table 3, so that w is a natural choice. For the other parameter, c provides a complementary perspective on sentiment dynamics: c and w describe the two transitions from the U compartment (to A or P, respectively). Using w and s would instead focus on transitions to the P compartment (from U and A, respectively), leaving out the important $U \to A$ transition.

Rearranging the sentiment conditions in Table 3 yields the sentiment endemism boundary curve:

$$f_A(c) = \left(\frac{b}{s+b}\right)c - b. \tag{58}$$

If $w < f_A(c)$, then the sentiment is endemic; if $w \geqslant f_A(c)$, then the sentiment goes extinct. The slope of eq. 58 is always positive, and the intercept is always negative, so $f_A(c)$ always has the same qualitative shape regardless of specific parameter value choices.

Our analysis of eq. 14 and eq. 15 demonstrates that $1 + v/b$ is greater than $1/[1-(v/(v+b))(w/(w+b))]$ and $1/[1-(v/(v+b))(s/(s+b)+(w-s)/c)]$. Thus, if $R_0 \geq 1 + v/b$, then the disease is always endemic (Table 3), and there is no disease endemism boundary curve.

In the case where $R_0 < 1 + v/b$, rearranging the disease conditions in Table 3 yields the disease endemism boundary curve:

$$f_I(c) = \begin{cases} \frac{vb}{\frac{v+b}{R_0} - b} - b & \text{if } c \leqslant \frac{v(s+b)}{\frac{v+b}{R_0} - b} \\ \left[\frac{v+b}{v}\left(1 - \frac{1}{R_0}\right) - \frac{s}{s+b}\right]c + s & \text{if } c > \frac{v(s+b)}{\frac{v+b}{R_0} - b}. \end{cases} \tag{59}$$

If $w < f_I(c)$, then the disease is endemic; if $w \geqslant f_I(c)$, then the disease goes extinct. In contrast to $f_A(c)$ (eq. 58), $f_I(c)$ (eq. 59) has different qualitative shapes for different sets of parameter values. We describe these sets in terms of R_0 for $R_0 > 0$.

The shape of $f_I(c)$ (eq. 59) has three components: the flat value, the linear slope, and the point of intersection between the flat and linear parts. In Figure 2C, the flat value is the value of w separating blue from orange, the linear slope is the slope of the line separating red from purple, and the point of intersection is the point where all four colors intersect. The flat value is negative for $R_0 \in (0,1)$ and zero for $R_0 = 1$. It grows without bound as R_0 increases from 1 to $1 + v/b$. The key value of R_0 here is 1.

The linear slope of $f_I(c)$ starts negative for $R_0 \in (0, 1/[1 - \frac{sv}{(s+b)(v+b)}])$, becomes zero for $R_0 = 1/[1 - \frac{sv}{(s+b)(v+b)}]$, and becomes positive for $R_0 > 1/[1 - \frac{sv}{(s+b)(v+b)}]$. The key value of R_0 here is $1/[1 - \frac{sv}{(s+b)(v+b)}]$, which is between 1 and $1 + v/b$. Finally, the point of intersection between the flat and linear parts of $f_I(c)$ is positive for $R_0 \in (0, 1 + v/b)$.

Combining these three components produces the four regimes in Figure 2. First, for $R_0 \in (0,1)$, the flat part of $f_I(c)$ is negative, the linear slope is also negative, and the point of intersection is positive. $f_I(c)$ does not appear in the positive part of the c-w plane. This situation appears in Figure 2A.

For $R_0 = 1$, the flat part of $f_I(c)$ is zero, the linear slope is negative, and the point of intersection is positive. For $R_0 \in (1, 1/[1 - \frac{sv}{(s+b)(v+b)}])$, the flat part of $f_I(c)$ is now positive, the linear slope is still negative, and the point of intersection is positive. This situation appears in Figure 2B. For $R_0 = 1/[1 - \frac{sv}{(s+b)(v+b)}]$, the linear slope of $f_I(c)$ becomes zero; in particular, $f_I(c) = s$.

For $R_0 \in (1/[1 - \frac{sv}{(s+b)(v+b)}], 1 + v/b)$, the flat part of $f_I(c)$ is positive, the linear slope is now positive, and the point of intersection is still positive. This situation appears in Figure 2C.

As $R_0 \to \infty$, for small s and small b, eq. 59 approaches eq. 58. Therefore, the more infectious the disease, the larger the portion of the parameter space where both disease and sentiment are stable or neither disease nor sentiment is stable. A larger portion of the parameter space consisting of either dual endemism or no endemism indicates a tighter coupling of disease and sentiment stability.

Appendix G. Numerical methods

For Figures 4-7, we used the `ode` function in the R package `deSolve` with step size 0.1 to numerically solve eq. 16. The initial condition for $(SU, SA, SP, IU, IA, IP, RU, RA, RP)$ in Figures 4-6 is $(0.998, 0.001, 0, 0.001, 0, 0, 0, 0, 0)$. For Figure 6, we solved until the time point for introducing the new disease case, then adjusted the compartment frequencies to introduce a new disease case by adding 0.001 to IU and removing 0.001 from SU, then again solving the system but with this adjusted set of compartment frequencies as the initial condition.

The initial value for Figure 7A is $(0.06375, 0.00525, 0, 0.001, 0, 0, 0, 0.06975, 0.86025)$. The initial condition for Figure 7B is approximately $(4.498 \times 10^{-5}, 0.0653, 4.483 \times 10^{-5}, 2.001 \times 10^{-8}, 1.952 \times 10^{-4}, 1.539 \times 10^{-7}, 3.502 \times 10^{-9}, 0.759, 0.175)$. Exact values can be obtained from eq. 12.

Appendix H. Assortative meeting

New rates of interaction. Assortative meeting changes the rates of interaction between susceptible and infected individuals. To describe the changes, consider random draws of two individuals from the population. Consider the infection of an undecided individual. This infection requires the draw of an SU individual and an I individual. Because U individuals are unaffected by assortativity, if the SU individual is drawn first, the rate of drawing an I individual is $I = IU + IA + IP$. If the I individual is drawn first, however, then under assortativity the only way to draw an SU individual is to have drawn an IU individual. This SU draw comes from the entire population because U is unaffected by assortativity. For the set of interactions that infect an SU individual, we have

$$\begin{aligned} 2(SU)I_U &= (SU)(IU + IA + IP) + (1-\alpha)(IU + IA + IP)(SU) + \alpha(IU)(SU) \\ &= (SU)(2(IU) + (2-\alpha)(IA + IP)) \\ I_U &= IU + \left(1 - \frac{\alpha}{2}\right)(IA + IP). \end{aligned}$$

Now consider the infection of an anti-vaccine individual. This infection requires the draw of an SA individual and an I individual. If the SA individual is drawn first, then under assortativity, an IA individual must be drawn from the A pool. If an IU individual is drawn first, assortativity has no effect. If an IA individual is drawn first, then under assortativity, the SA individual must be drawn from the A pool. If an IP individual is drawn first, then an SA individual cannot be drawn under assortativity. We have

$$\begin{aligned} 2(SA)I_A &= (SA)\left[(1-\alpha)(IU + IA + IP) + \alpha\frac{IA}{A}\right] + (IU)(SA) + (IA)\left[(1-\alpha)(SA) + \alpha\frac{(SA)}{A}\right] \\ &\quad +(IP)(1-\alpha)(SA) \\ &= (SA)\left[(2-\alpha)(IU) + \left(2\left(1 - \alpha + \frac{\alpha}{A}\right)\right)(IA) + 2(1-\alpha)(IP)\right] \\ I_A &= \left(1 - \frac{\alpha}{2}\right)(IU) + \left(1 + \alpha\frac{1-A}{A}\right)(IA) + (1-\alpha)(IP). \end{aligned}$$

Because assortativity works in the same way for pro-vaccine individuals as it does for anti-vaccine individuals, infection of a pro-vaccine individual is analogous to the infection of an anti-vaccine individual, except the roles of A and P are switched.

$$\begin{aligned} 2(SP)I_P &= (SP)\left[(1-\alpha)(IU + IA + IP) + \alpha\frac{IP}{P}\right] + (IU)(SP) + (IA)(1-\alpha)(SP) \\ &\quad +(IP)\left[(1-\alpha)(SP) + \alpha\frac{(SP)}{P}\right] \\ &= (SP)\left[(2-\alpha)(IU) + 2(1-\alpha)(IA) + \left(2\left(1 - \alpha + \frac{\alpha}{P}\right)\right)(IP)\right] \\ I_P &= \left(1 - \frac{\alpha}{2}\right)(IU) + (1-\alpha)(IA) + \left(1 + \alpha\frac{1-P}{P}\right)(IP). \end{aligned}$$

Changes to equilibria. In the assortative meeting model (eq. 16), analytical equilibria for the SFE and the EE are not feasible to obtain. The DSFE is unchanged (eq. 6) and the DFE is now:

$$\begin{aligned}\widehat{SU} &= \frac{s+b}{c\left(1-\frac{\alpha}{2}\right)}\\ \widehat{SA} &= \frac{b}{s+b} - \frac{w+b}{c\left(1-\frac{\alpha}{2}\right)}\\ \widehat{SP} &= \frac{bs}{(s+b)(v+b)} + \frac{b(w-s)}{c\left(1-\frac{\alpha}{2}\right)(v+b)}\\ \widehat{RP} &= \frac{vs}{(s+b)(v+b)} + \frac{v(w-s)}{c\left(1-\frac{\alpha}{2}\right)(v+b)}.\end{aligned}$$

Increasing assortativity therefore increases the equilibrium frequency of the SU class and decreases that of the SA class. These results accord with the expectation that assortativity makes sentiment more difficult to transmit, because $A \times U$ interactions decrease in frequency in favor of $A \times A$ interactions.

The sentiment stability boundary from eq. 58 becomes

$$w = \frac{b}{s+b}\left(1-\frac{\alpha}{2}\right)c - b.$$

Increasing assortativity decreases the slope of this line, decreasing the area of parameter space where sentiment is endemic.

Initial rate of increase. We now investigate the effect of assortativity when introducing an infected individual into a population without endemic disease. The quantity $\frac{dI}{dt} = I'$ determines the rate of change of disease frequency. We are interested in how adding a single infected individual to the population affects how quickly the disease increases (or decreases). If we add an infected individual of sentiment type $i = U, A, P$, then the "direction" we perturb the system is given by

$$\begin{aligned}v_U &= \left(-\frac{1}{2}, 0, 0, \frac{1}{2}, 0, 0, 0, 0, 0\right)\\ v_A &= \left(0, -\frac{1}{2}, 0, 0, \frac{1}{2}, 0, 0, 0, 0\right)\\ v_P &= \left(0, 0, -\frac{1}{2}, 0, 0, \frac{1}{2}, 0, 0, 0\right).\end{aligned} \tag{60}$$

The direction vectors v_U, v_A, and v_P in eq. 60 correspond to transferring a small frequency of susceptible individuals of type i to infected individuals of type i. They therefore represent the infection of an individual from either the SU, SA, or SP classes, respectively, in the limit of an infinite population.

To obtain the effect that perturbing the system in direction v_i (eq. 60) has on the speed at which the disease frequency increases or decreases, we compute the directional derivative $D_i I'$ of I' with respect to the vector v_i for $i = U$, A, and P, respectively. For instance, increasing $D_U I'$ increases the rate that the disease spreads if an IU individual is introduced in the population.

Computing directional derivatives with respect to the three vectors in eq. 60 requires the gradient of I'. Because the last three terms in the vectors are all zero, we only need the first six partial derivatives:

$$\begin{aligned}
\frac{\partial I'}{\partial(SU)} &= r\left((IU) + \left(1-\frac{\alpha}{2}\right)(IA+IP)\right)\\
\frac{\partial I'}{\partial(SA)} &= r\left[\left(1-\frac{\alpha}{2}\right)(IU) + (1-\alpha)(IP) + (IA)\left(1+\alpha\frac{1-A}{A} - \alpha\frac{(SA)}{A^2}\right)\right]\\
\frac{\partial I'}{\partial(SP)} &= r\left[\left(1-\frac{\alpha}{2}\right)(IU) + (1-\alpha)(IA) + (IP)\left(1+\alpha\frac{1-P}{P} - \alpha\frac{(SP)}{P^2}\right)\right]\\
\frac{\partial I'}{\partial(IU)} &= r\left[SU + \left(1-\frac{\alpha}{2}\right)(SA+SP)\right] - (g+b)\\
\frac{\partial I'}{\partial(IA)} &= r\left[\left(1-\frac{\alpha}{2}\right)(SU) + (1-\alpha)(SP) + (SA)\left(1+\alpha\frac{1-A}{A} - \alpha\frac{(IA)}{A^2}\right)\right] - (g+b)\\
\frac{\partial I'}{\partial(IP)} &= r\left[\left(1-\frac{\alpha}{2}\right)(SU) + (1-\alpha)(SA) + (SP)\left(1+\alpha\frac{1-P}{P} - \alpha\frac{(IP)}{P^2}\right)\right] - (g+b).
\end{aligned} \tag{61}$$

The directional derivative is the dot product of the gradient of I' (eq. 61) with a direction vector v_i (eq. 60). The first three partial derivatives in eq. 61 are all zero when evaluated at a disease-free starting condition, so the sign of the directional derivative is determined by the sign of the last three partial derivatives in eq. 61.

Once we have the directional derivatives, we can analyze the effect that increasing α has on the effect of adding a new infection case by observing the sign of the coefficient of α in the directional derivative. If the sign is positive, then assortativity increases the effect of a new infection, and if the sign is negative, then assortativity decreases the effect of a new infection.

We focus here on disease-free situations so that we can directly study the effect of α on the rate of increase of the disease without also studying the effect of α on the equilibrium frequency of the disease in the first place, as the initial increase rate depends on the equilibrium frequency.

For v_U, the infection occurs in the SU population. The directional derivative is

$$D_U I' = \frac{r}{2}\left(S - \frac{\alpha}{2}[S-(SU)] - \frac{1}{R_0}\right).$$

Thus, the rate of initial increase of the epidemic decreases with increasing assortativity when the epidemic starts with an undecided individual.

For v_A, the infection occurs in the SA population. The directional derivative is

$$D_A I' = \frac{r}{2}\left[S + \alpha\left(\frac{(SA)}{A} - S + \frac{1}{2}(SU)\right) - \frac{1}{R_0}\right].$$

For both a new disease ($S=1$) and at the DFE ($S<1$), $A=(SI)$, so this equation becomes

$$D_A I' = \frac{r}{2}\left[S + \alpha\left(1 - S + \frac{1}{2}(SU)\right) - \frac{1}{R_0}\right].$$

Assortativity increases the initial rate of increase when the epidemic starts in an anti-vaccine individual.

For v_P, the infection occurs in the SP population. The directional derivative is

$$D_P I' = \frac{r}{2}\left[S + \alpha\left(\frac{(SP)}{P} - S + \frac{1}{2}(SU)\right) - \frac{1}{R_0}\right].$$

In the case of a new disease ($S=1$), $P=(SP)$, so we have

$$D_P I' = \frac{r}{2}\left[1 + \alpha\left(1 - S + \frac{1}{2}(SU)\right) - \frac{1}{R_0}\right].$$

Assortativity also increases the initial rate of increase of a new disease when the epidemic starts in a pro-vaccine individual in the case of a new disease. However, at the DFE, we have

$$D_P I' = \frac{r}{2}\left[S + \alpha\left(\frac{(SP)}{(SP)+(RP)} - S + \frac{1}{2}(SU)\right) - \frac{1}{R_0}\right]. \tag{62}$$

At the DFE, $\frac{SP}{SP+RP} = \frac{b}{v+b}$, so the condition for the sign of α to be positive is

$$\frac{b}{v+b} \geqslant S - \frac{1}{2}(SU).$$

This condition reduces to

$$1 \leqslant \frac{(s+b)[(s+b)(v+b)-2sv]}{2bv(c-w)}.$$

For $s=0$, it becomes

$$1 \leqslant \frac{b(v+b)}{2v(c-w)}.$$

At the DFE, with $s=0$, $c-w \geqslant b$, so we have

$$\frac{b(v+b)}{2v(c-w)} \leqslant \frac{1}{2} + \frac{b}{2v}. \tag{63}$$

A requirement for the condition eq. 63 is $b \geqslant v$, which is unlikely in practice. We therefore expect the coefficient of α in eq. 62 to be negative; assortativity in general decreases the rate of initial increase of an epidemic at the DFE when the epidemic starts in a pro-vaccine individual.

References

Gantmacher, F. (1960). *The Theory of Matrices, Vol. 2.* Chelsea.

Guckenheimer, J., & Holmes, P. (1990). *Nonlinear Oscillations, Dynamical Systems, and Bifurcations of Vector Fields.* Springer.

Meinsma, G. (1995). Elementary proof of the Routh–Hurwitz test. *Systems Control Letters*, *25*(4), 237–242.
